# Link between Spin–Orbit Relativity and Magnetically Induced Current Densities in Heavy-Atom Hydrides: *trans*-Ligand Influence

**DOI:** 10.1021/jacsau.6c00346

**Published:** 2026-06-02

**Authors:** Daniel Blasco, Jan Novotný, James R. Asher, Raphael J. F. Berger, Stanislav Komorovsky, Radek Marek

**Affiliations:** † CEITEC − Central European Institute of Technology, 37748 Masaryk University, Kamenice 5, 62500 Brno, Czechia; ‡ Department of Chemistry, Faculty of Science, 37748 Masaryk University, Kamenice 5, 62500 Brno, Czechia; § Institute of Inorganic Chemistry, 231829 Slovak Academy of Sciences, Dúbravská cesta 9, 84536 Bratislava, Slovakia; ∥ Department of Chemistry and Physics of Materials, 27257 Paris Lodron University of Salzburg, Jakob-Haringerstraße 2A, 5020 Salzburg, Austria

**Keywords:** electronic motion, spin−orbit coupling, magnetically induced current density, Dirac–Kohn−Sham level, trans-ligand influence

## Abstract

Interactions between individual atoms underpin the structure and behavior of matter. These interactions govern atomic positions and dynamics, as well as the organization of electrons—particularly in the frontier region. Because electrons lie at the core of chemical phenomena, numerous theoretical frameworks have been developed to rationalize the molecular structure and properties. Electronic motion within molecules and the resulting induced currents provide powerful probes of the molecular or supramolecular structure, building on and going beyond molecular orbital and valence bond theories. In particular, current density offers a spatially-resolved description of the electronic response to external perturbations, enabling direct analysis of electron delocalization and magnetic response in molecular systems. In this work, the effect of relativistic spin–orbit (SO) coupling on the strength and topology of the magnetically induced current density (MICD) is analyzed in depth for a series of model heavy-atom hydrides at the four-component Dirac-Kohn–Sham level. For the most simple molecules, TlH, HAt, and AuH, we demonstrate a connection between the SO effects on the molecular geometry, strength and topology of MICDs, and ligand ^1^H NMR shielding. For model **HMX** molecules, where M = Au^I^, Hg^II^; X = F, Cl, Ph, CH_3_, H, SiH_3_, BH_2_, the hydride deshielding due to the slight elongation of the M–H bond upon increasing the *trans*-ligand influence (TLI) of X is shown to be marginal when compared to that originating from the electronic SO effect. In particular, the inclusion of SO effects gives rise to highly localized paratropic MICD vortices on the hydride position of those complexes bearing strong TLI ligands. Our results disprove the previously proposed governing role of the current around the metal atom (similar to the classical Buckingham-Stephens model for transition metal hydrides) associated with TLI-induced variations in the metal–hydrogen bond length in determining the characteristic ligand ^1^H NMR shifts.

## Graphical abstract

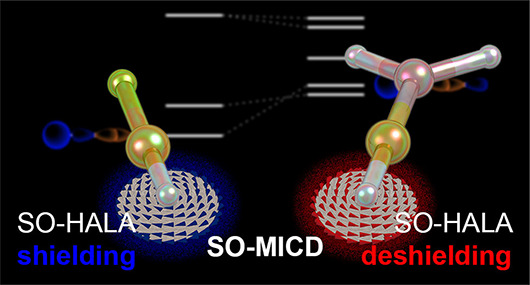

## Introduction

The magnetically induced current density [MICD; $J_{\alpha }^{B_{\beta }}\left(r\right)$ ] is a vector field describing the induced flow of the electron current in the α direction when a small external magnetic field **B** is applied in the β direction, with α, β ∈ {*x*, *y*, *z*}.^1–3^ It is commonly employed to assess the aromatic character of molecular rings according to the magnetic criterion of aromaticity,^4,5^ see Figure 1. However, it is also an important quantum mechanical entity which can be used to calculate any magnetic response property, e.g. via the Biot-Savart law.^3,6^ Because of this, the calculation, visualization, and interpretation of the MICD provides a connection between experiments and theory, because measurable nuclear magnetic resonance (NMR) parameters can be derived from the MICD and related to the electron delocalization pathways in molecules.^2^

**Figure 1:**
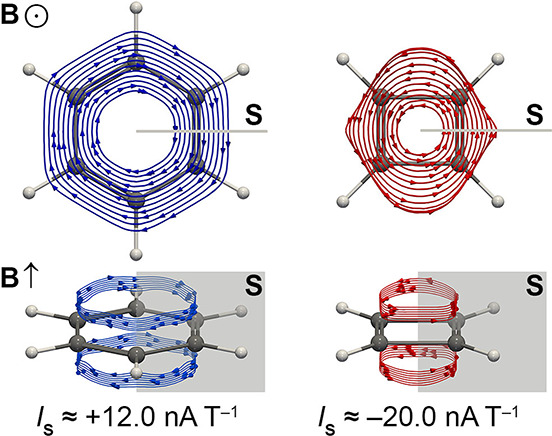
Sign and magnitude of the MICD strength obtained by plane integration (*I*_**S**_) can be used to discern between aromatic (*I*_**S**_ > +6.0 nA T^–1^), nonaromatic (*I*_**S**_ ≈ 0.0 nA T^–1^), and antiaromatic (*I*_**S**_ < −6.0 nA T^–1^) molecular rings.^7,8^ The archetypal aromatic and antiaromatic molecules benzene (left) and cyclobutadiene (right) sustain global diatropic (blue streamlines) and paratropic (red streamlines) MICDs, respectively. Note the different direction of rotation w.r.t. the external magnetic field vector of the MICDs.

Although the relativistic spin–orbit-induced (SO) effect of a heavy atom (HA) on the NMR shielding constant of a bonded light atom (LA; SO-HALA effect) is nowadays very well-known and has a sound theoretical background,^9–13^ its corresponding effect on the strength and topology of the MICD has not yet been studied in the same depth. This research field was pioneered by Berger et al., who showed that SO coupling (SOC) enhances the curvature of the MICD and gives rise to distortions at the hydride position of the model d-block metal hydrides AuH and HgH_2_.^14^ The direction of rotation of the SO-induced distortions of the MICD (SO-MICD) w.r.t. the external magnetic field vector, that is, their tropicity, can be related to the SO-HALA (de)­shielding of H. An attempt to rationalize the SO-MICD tropicity in terms of the shape of the highest-lying NMR active molecular orbitals (MOs) was proposed later on, suggesting that diatropic SO-MICD vortices appear only between MO lobes.^15^ Most of the studies regarding relativistic SO effects in the magnetic response properties of d-block metal compounds have focused on analyzing their role in the aromaticity descriptors of metal-containing (anti)­aromatic molecular rings and all-metal clusters.^7,16–18^

Given that electron distribution and interatomic electron sharing constitute the essence of chemical bonding,^19–21^ MICDs offer a direct means of probing bonding interactions.^22^ Previous studies have shown that SOC effects around the HA influence the character of HA–LA bonding.^23^ In addition, the HA–LA bond may be modulated by an additional ligand (X) coordinated to the HA in the position *trans* to the LA, a phenomenon referred to as the *trans*-ligand influence (TLI).^24–26^ The structural component of the TLI involves geometric alterations (changes in the HA–LA bond lengths) arising from the competition between two *trans* ligands (LA and X) for bonding to the central metal atom (M).^11,13,27^ In other words, the TLI is a thermodynamic effect connected with the modulation of spectroscopic parameters of a ligand atom (here H) in a metal complex by the σ-donor strength of its *trans* ligand (here X). This effect is particularly evident for the ^1^H NMR signals of transition-metal (TM) hydrides, which resonate at characteristic negative shifts in the ^1^H NMR spectrum and are readily affected by subtle structural and/or electronic changes. According to the classical Buckingham-Stephens model,^28,29^ the electrons of the partially filled valence d-shells of TMs sustain local paratropic currents that further shield the otherwise not-so-shielded hydride. More importantly for the purposes of this paper, the distance of H from the TM, which in turn depends on the TLI strength, determines its degree of shielding. The paratropic character of the TM atomic vortex has been inferred by examining the anisotropy of the NMR shielding tensor of bonded H in a series of early TM carbonyl complexes.^30^ However, it has also been demonstrated that the inclusion of SOC effects is necessary for a proper description of the phenomenon, especially for sixth-row late TM complexes in which SOC effects are significant.^11,15,31–36^ These studies have also demonstrated that the magnitude of SO-HALA is modulated by the degree of bond covalency, as a result of competition between the mutually *trans*-oriented ligands for bonding to the same TM d orbital.^33,34^

Because elements of the NMR shielding tensor (σ) of a probe nucleus K can be calculated from $J_{\alpha }^{B_{\beta }}\left(r\right)$ ,^6,37,38^ the SO-HALA effect on σ^K^ must also be reflected in the SO-MICD that circulates around K. Herein we provide further insights into the various effects of SOC on the magnetic response properties, and particularly the strength and topology of the MICD circulating around the hydrogen atom, of selected model HA hydrides. To show the consistency of the connection, we have considered the simple diatomic molecules TlH, AtH, and AuH, and two series of linear model hydrido-complexes **HMX** of general formula [HMX]^q^ or [HML]^q+1^, where M­(q) = Au^I^(1−) (**1**), and Hg^II^(0) (**2**); X = F (**a**), Cl (**b**), Ph (**c**), CH_3_ (**d**) H (**e**), SiH_3_ (**f**), and BH_2_ (**g**); and L = NH_3_ (**h**), CO (**i**), and PH_3_ (**j**); for molecular structures, see Figure 2. The paper is organized as follows. First, the details of the computational methods employed are stated. Then, in the first part of Results and Discussion, we calculate bond strength descriptors and correlate the massive SO effects on the magnetic response properties of TlH and AtH with the topology of their MICDs. In the second part of Results and Discussion, we examine the dependence of the NMR shielding constants and MICD strengths of **HMX** complexes on M–H distances, along with the selective inclusion or exclusion of SOC effects. Finally, conclusions and perspectives are given.

**Figure 2:**
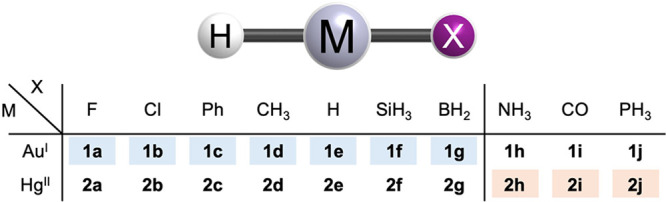
Structure of model **HMX** molecules. Molecules shaded in blue are negatively charged, whereas molecules shaded in orange are positively charged.

## Computational Details

### Molecular Structure Optimization

The molecular structure optimizations were carried out with ADF 2024.102.^39^ The molecular structures were optimized at the density functional theory (DFT) level with the PBE0 functional^40–42^ and a combination of all-electron Slater-type core triple-ζ and valence quadruple-ζ with four polarization functions (QZ4P) basis sets on the HA and triple-ζ with two polarization functions (TZ2P) basis sets on the rest of the LAs.^43,44^ Scalar relativistic (SR) effects were considered by using the zeroth-order regular approximation (ZORA) Hamiltonian.^45–47^

### Fully Relativistic Four-Component Calculations

The four-component relativistic Dirac–Kohn–Sham (4c-DKS) calculations were carried out with ReSpect.^48^ The PBE0 functional was used^40–42^ together with the uncontracted Dyall-type quadruple-ζ (dyall-vqz) basis sets^49–51^ for the HA and uncontracted Jensen-type pcS-3 (upcS-3) basis sets^52,53^ for the rest of the LAs.

The 4c-DKS MICD was calculated using theory from ref 54. The strength of the MICD circulating around H (*I* ^H^) was obtained by numerical integration of the MICD on a 6 Å × 6 Å regular grid formed by 121 × 121 evenly spaced points. The grid was placed parallel to the HA–H bond with H placed in the center of one of the sides (see Figure 3). The external magnetic field vector was set parallel to the integration plane and perpendicular to the principal axis of the molecule. The sign of the integrated MICD strength indicates its tropicity; it is positive if it flows in the diatropic (classical) direction or negative if it does so in the paratropic (nonclassical) direction. It should be stressed that the partition of the MICD into diatropic and paratropic components refers only to their tropicity and that it is not analogous to the partition of the NMR shielding constants into diamagnetic and paramagnetic components.

**Figure 3:**
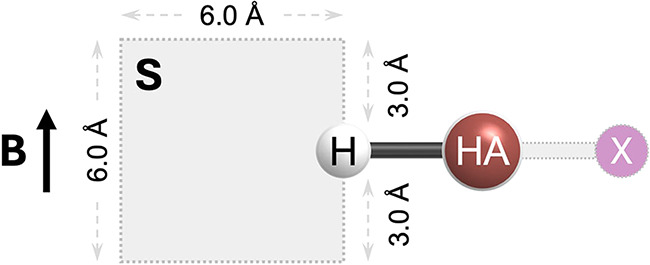
Dimension and position of the integration plane **S** w.r.t. the heavy-atom (HA) molecule and the external magnetic field vector **B**.

To scale the SOC contribution to 4c-DKS, the 1–3 complex quaternion constituents of the Fock matrix were multiplied by a scaling factor λ. The specific SOC contributions to NMR shielding constants (σ), MICDs [ $J_{\alpha }^{B_{\beta }}\left(r\right)$ ], and MICD strengths (*I* ^H^), were approximated to the difference between two identical calculations in which the SOC contributions were scaled by λ = 1 (SOR-4c-DKS) and λ = 0 (SR-4c-DKS).

Bond dissociation energies (BDEs) were computed for the homolytic fragmentation HX → H + X as the difference between the total electronic energy of the molecule at its equilibrium distance (*r*_e_) and the sum of the energies of the free neutral atoms,

$$\mathrm{BDE}=E^{\mathrm{HX}}(r_{e})-E^{H}-E^{X}$$ 1

To extract bond force constants, the ab initio electronic energies *E* ^HX^(*r*_*i*_) computed on a grid {*r*_*i*_}_*i*=1_^*N*^ at the 4c-DKS level were analyzed in terms of the Morse model. We first introduce the standard Morse potential

$$V_{M}(r)=D_{e}(1-\exp[-a(r-r_{e})]{)}^{2}$$ 2

which upon expansion and by choosing the dissociation limit as the zero reference for energy yields the shifted form

$$U_{M}(r;D_{e},a,r_{e})=D_{e}(\exp[-2a(r-r_{e})]-2\exp[-a(r-r_{e})])$$ 3

The Morse parameters **θ** = (*D*_e_, *a*, *r*_e_) were then determined by weighted nonlinear least-squares fitting,

$$U(r_{i})\overset{\mathrm{LSQ}\;\mathrm{fit}}{\underset{\theta =(D_{e},a,r_{e})}{\to }}\hat{\theta }=({\hat{D}}_{e},\hat{a},{\hat{r}}_{e})$$ 4

with

$$({\hat{D}}_{e},\hat{a},{\hat{r}}_{e}):=\arg\min_{D_{e}>0,a>0}\sum_{i=1}^{N}w_{i}(U_{i}-U_{M}(r_{i};D_{e},a,r_{e}){)}^{2}$$ 5

where *w*_*i*_ are optional weights (e.g., chosen to emphasize the near-equilibrium region). Within the Morse model, the harmonic bond force constant follows from the curvature at equilibrium,

$$k_{e}:=\frac{d^{2}V_{M}(r)}{dr^{2}}{|}_{r=r_{e}}=2D_{e}a^{2},\;\mathrm{so}\;\mathrm{that}\;a=\sqrt{\frac{k_{e}}{2D_{e}}}$$ 6

### Magnetic Shielding Densities

The 4c-DKS magnetic shielding density ( $S^{H}$ , MSD) of H was computed according to the theory presented in the appendix of ref 54 (in Hartree atomic units)

$$\sigma_{uv}^{H}\overset{\mathrm{def}}{=}\int S_{uv}^{H}dV={-\frac{1}{c}\int \frac{d\overset{\to }{j}}{dB_{u}}|}_{0}\cdot \frac{d\overset{\to }{A}}{d\mu_{v}^{H}}dV,where\;\;\overset{\to }{A}=\frac{{\overset{\to }{\mu }}^{H}\times {\overset{\to }{r}}_{H}}{r_{H}^{3}}$$ 7

where *c* is the speed of light, *u* and *v* are Cartesian indices, $\vec{j}$ represents the electronic current density, ${\vec{r}}_{H}$ is the electron position relative to the position of H, ${\vec{\mu }}^{H}$ is the nuclear magnetic moment of H, and σ_*uv*_^H^ represents the NMR shielding tensor of H.

The atomic partition of the MSD of H was calculated by numerical integration over atomic domains with the gauge-including magnetically induced currents (GIMIC) method.^2,55,56^ For this, the unperturbed and magnetically-perturbed one-electron density matrices were calculated with Turbomole 7.8-β ^57,58^ at the scalar-relativistic all-electron exact two-component (sc-X2C) DFT level with the PBE0 functional^40–42^ and x2c-QZVPall-s basis sets^59^ on all atoms. This level of theory is an approximation to SR-4c-DKS. The partition cannot be calculated at the full X2C level, that is, with inclusion of SO interaction, because only scalar-relativistic X2C is implemented within the GIMIC program.

### Third-Order Perturbation Analysis

The SO contributions to the Fermi-contact interaction (FC) mechanism of SO-HALA (σ^SO/FC^) were calculated using the third-order perturbation analysis (PT3) as implemented^11,12^ in ReSpect.^48^ For analysis of MO contributions to σ^SO/FC^, a common gauge origin was used and dyall-vdz basis sets^49–51^ for the HA and upcS-1 basis sets^52,53^ for the rest of the LAs were chosen. The atomic orbital (AO) composition of the active nonrelativistic MOs was obtained by performing a Mulliken population analysis at the same level of theory using the ReSpect program.^48^

### Visualization

The nonrelativistic MOs and the 4c-DKS charge densities were visualized and plotted with VMD.^60^ The total SO electron deformation densities (SO-EDD), SO and magnetically induced spin densities (SOM-ISD), MSDs, and MICD and SO-MICD delocalization pathways were visualized and plotted with ParaView 5.11.0.^61^

## Results and Discussion

### Diatomic TlH and HAt Systems Revisited

#### Bond Dissociation Energies and Force Constants

In previous studies, TlH and HAt were considered paradigmatic models exhibiting large and opposite SO-HALA effects due to the presence of vacant and occupied HA-centered 6p orbitals in the nonrelativistic limit, respectively.^12,23^ For completeness, we recall that SOC leads to a contraction of 3.3 pm of the Tl–H bond and a proton deshielding of −189 ppm, and to a lengthening by 2.9 pm of the At–H bond and a proton shielding of +32 ppm. The notable SO-induced changes in the bond lengths intuitively suggest the existence of an analogous effect in the related bond dissociation energies (BDE) and force constants (*k*_e_). Thus, one would expect a stronger bond, i.e. larger BDE and *k*_e_ when SOC shortens it, as in the case of TlH. To verify this assumption, we have calculated the BDE and *k*_e_ of model diatomic HA hydrides exhibiting large negative (TlH), large positive (HAt), and negligible (AuH) SO effects on bond lengths at the 4c-DKS level of theory. The results are summarized in Table 1.

**Table 1:** SO Contribution (Δ) to Equilibrium Bond Lengths (*r*_e_ in pm), Homolytic BDEs (in kcal mol^–1^), and Force Constants (*k*_e_ in N m^–1^) of Molecules TlH, HAt, and AuH

molecule	Δ*r*_e_	Δ(BDE)	Δ*k*_e_
TlH	–3.4	+12.23	+2 ± 4
HAt	+2.7	+15.89	–44 ± 4
AuH	–0.1	–0.85	+3 ± 8

Inspection of Table 1 reveals that there is no clear relationship between SO contributions to BDEs and SO modulations of bond lengths. Instead, the inclusion of SO effects weakens the more strongly affected bonds, regardless of whether they are lengthened or shortened. For AuH, which is only slightly affected by SOC, the stabilization provided is negligible. We note that a severe limitation of BDEs is that the reference state for the fragments is not clear.^62^ If heterolytic bond splitting patterns are considered, the resulting BDEs vary drastically, even changing their sign (see Table S1).^63^ In the absence of experimental evidence of the preferred reactivity of the selected hydrides, the choice of one bond splitting pattern over the others is somewhat arbitrary. On the other hand, the use of force constants has the advantage that they only depend on the equilibrium structure of the investigated molecule, although their relation to bond strengths is not causal, with numerous exceptions to the correlation between them.^64^ The only significant decrease of *k*_e_ is observed for HAt, whereas for TlH and AuH, the slight SO-induced increase of *k*_*e*_ is of the size of the fitting error. The plots showing the fitting of the total electronic energy as a function of distance for TlH, HAt, and AuH are presented in Figure S1. An analysis of the Tl–H bond revealed that the SO-induced coupling of the highest-lying occupied Kramers pair with the lowest-lying virtual one induces electron depopulation of the σ bond in favor of Tl-centered nonbonding orbitals.^65^ This apparent contradiction, namely bond shortening accompanied by a decrease in bond covalence, can be explained with purely electrostatic arguments.^23^ In contrast, the SOC effect on the At–H bond length and its covalency behaves as expected, i.e. the At–H bond is weakened by SOC because it induces population of σ antibonding orbitals from At-centered nonbonding ones (vide infra).

#### MICD Strengths

We have calculated the strength of the MICD circulating around H at the SR-4c-DKS (*I*_SR_^H^) and SOR-4c-DKS (*I*_SOR_^H^) levels of theory by plane integration (see Figure 3). The SOC contribution to the MICD strength (Δ*I*_SO_^H^) is calculated as the difference between the values at the SOR-4c-DKS and SR-4c-DKS levels. The MICD and SO-MICD strengths for molecules TlH, HAt, and AuH are collected in Table 2, together with the SO-HALA NMR shielding constants for purposes of comparison.

**Table 2:** Net MICD Strength Circulating around H (*I* ^H^ in nA T^–1^) Calculated at the SR-4c-DKS and SOR-4c-DKS Levels of Theory, and the SO Contributions to the MICD Strength (Δ*I*_SO_^H^ in nA T^–1^) and to the Total ^1^H NMR Shielding Constant (Δσ_SO_^H^ in ppm) of the Molecules TlH, HAt, and AuH

molecule	*I*_SR_^H^	*I*_SOR_^H^	Δ*I*_SO_^H^	Δσ_SO_^H^
TlH	+1.54	–13.56	–15.10	–188.7
HAt	+5.91	+9.23	+3.32	+32.4
AuH	+5.96	+7.76	+1.80	+20.2

The inclusion of SOC leads to a noticeable strengthening of the MICDs, as the absolute values of *I* ^H^ at the SOR-4c-DKS level are larger than the respective ones at the SR-4c-DKS level, see Table 2. However, whereas the tropicity of the current circulating around H is preserved in HAt and AuH, it is reversed in the case of TlH. This striking change of tropicity in TlH is better understood when visualizing the MICD delocalization pathways (vide infra). There is an agreement between the sign and magnitude of Δσ_SO_^H^ and Δ*I*_SO_^H^, suggesting a connection between them.^13^

#### MICD Topologies

The MICD delocalization pathways of TlH and HAt are shown in Figure 4. For a discussion of the MICD pathways of AuH, see ref 14. For TlH, the delocalization pathways are surprisingly complicated even at the SR level. There are two diatropic vortices centered on Tl and H, and a large paratropic vortex in the bonding space between them. A similar MICD topology has been identified as a common feature of closed-shell diatomic molecules with four valence electrons such as BH, CH^+^ and BeH^–^, and appears to be related to their calculated induced orbital paramagnetism.^66^ At the SOR level, the paratropic bond and diatropic H vortices are merged into one large out-of-plane vortex centered on the H nucleus, and Tl now features an area of poloidal currents on the side opposite the Tl–H bond, see Figure S2. For HAt at the SR level, a global diatropic current surrounds the entire molecule and flows smoothly through H. Independent vortices are not observed. However, the inclusion of SO effects gives rise to a drop-shaped diatropic vortex roughly centered at the position of H and pointing toward At. The new vortex is embedded in the global current, leading to the global diatropic pathways being pushed away from H. Our observations can be considered further indication of the SO-induced covalent contribution to the bond in H–At, as well as of the SO-induced electrostatic contribution in Tl–H.

**Figure 4:**
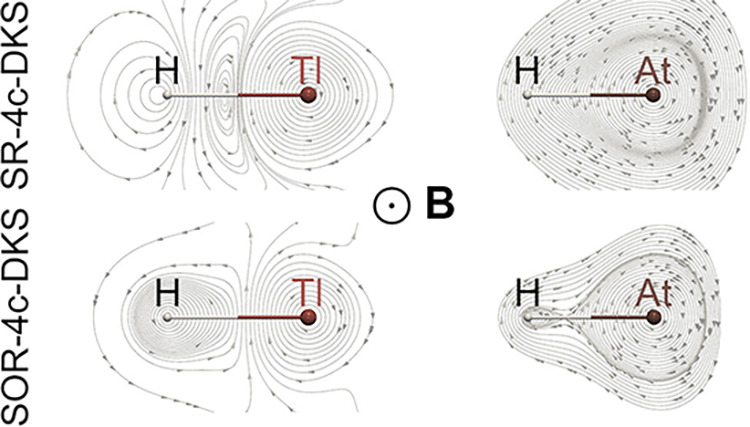
MICD delocalization pathways of TlH (left) and HAt (right) at the SR-4c-DKS (top) and SOR-4c-DKS (bottom) levels of theory. The arrowheads indicate the direction of the MICD.

Berger et al. demonstrated that the SO-MICD, up to first order in the SO interaction, is proportional to minus the curl of the spin density (SD) that arises due to the presence of both the external magnetic field and SOC (SO and magnetically induced SD, SOM-ISD).^12,14^ The topology of the SO-MICD of TlH and HAt is shown in Figure S3. Some of us have demonstrated a solid connection between the signs of SO-HALA and SOM-ISD (and to a large extent also with that of SO electron deformation density, SO-EDD).^11,12,23^ To show that this connection extends to the tropicity of the entire SO-MICD, we depict in Figure 5 the out-of-plane component of SOM-ISD in the molecular plane together with the tropicity of SO-MICD, for TlH and HAt. Clearly, the tropicity of the SO-MICD is in very good agreement with the sign of the SOM-ISD. Thus, SO-MICD is diatropic in the regions of induced α-spin density (or β-spin depletion, in blue) and paratropic in the regions of induced β-spin density (or α-spin depletion, in red). However, there is a mismatch in certain areas close to the heavy atoms, e.g. the SO-MICD on the rear part of the β-spin back lobe of Tl has the same tropicity as the surrounding α-spin lobes despite having opposite induced spin densities. We conclude that such a relation may hold only in regions where second-order SO effects are negligible, and thus where SOC contributes only to the spin current, which is expressed as the curl of the spin density.^14^

**Figure 5:**
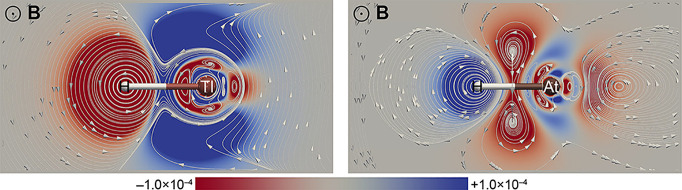
Out-of-plane component of the SOM-ISD superimposed with the direction of the SO-MICD for molecules TlH (left) and HAt (right). The white arrowheads indicate the direction of the SO-MICD.

#### Molecular Orbital Contributions to the MICD

The MO picture obtained with 4c-DKS methodology does not provide a chemically intuitive representation of the origin of SO-HALA, as the electronic structure is described in terms of complex spinors that defy easy visualization. This issue can be overcome by applying perturbation theory based on nonrelativistic MOs,^11^ provided that the perturbational treatment of SOC adequately reproduces the studied trends in the 4c-DKS results. In the framework of the third-order perturbation theory (PT3) formalism, three perturbative operators are involved: spin–orbit coupling (SOC), Fermi-contact interaction (FC), and external magnetic field–angular momentum coupling (L). As a result, the spin–orbit/Fermi contact term (σ^SO/FC^), which is the trend-defining contribution to the SO-HALA shift, can be decomposed into contributions from triads of MOs linked by the three aforementioned operators, which can involve two occupied and one virtual MOs (△ coupling), or one occupied and two virtual MOs (▽ coupling). The mathematical expressions for σ^△^ and σ^▽^ can be found in ref 11.

For TlH, the main contribution to the giant SO deshielding was shown to originate from the △ coupling between the σ-bonding HOMO and the doubly-degenerate nonbonding HA-centered LUMO,^13,23^ see Figure 6, top left. For HAt, the σ-bonding HOMO–1, the doubly-degenerate nonbonding HA-centered HOMO, and the σ-antibonding LUMO are involved in two different △ coupling schemes differing in the arrangement of the operators, see Figures 6, bottom left, and S5. Both schemes of HAt have a shielding character. The (de)­shielding character of these couplings is also observed in the value of the MICD strength originating from the involved MOs exclusively. We ran calculations increasing the SO scaling factor λ from 0.0 (SR-4c-DKS) to 1.0 (SOR-4c-DKS) in steps of 0.2 to observe the effect of SOC on the MO energies, charge densities, and MICD strengths of TlH and HAt. Figures S4 and S6 show the trends in energy and charge-density for TlH and HAt, respectively, whereas Tables S5 and S6 collect the total MICD strength and that originating only from the dominant PT3 coupling as a function of λ. SOC breaks the degeneracy of the nonbonding LUMO (TlH) or HOMO (HAt) orbitals by admixing H β-spin density into only one of the two α-spinors (and vice versa for the β-spinors; see Tables S3 and S4). Due to this spin imbalance, the PT3 coupling becomes stronger with larger λ, as reflected in the progressive increase of the associated MICD strength, which becomes more diatropic for HAt and paratropic for TlH. The right-hand side of Figure 6 shows the SOM-ISD and SO-MICD associated with the MOs involved in the PT3 couplings shown on the left-hand side of the figure. A comparison with the total densities shown in Figure 5 demonstrates that the leading PT3 couplings also capture the most salient features of SOM-ISD and SO-MICD, namely the sign and tropicity of the current vortex on H and the overall distribution of the SOM-ISD. Again, the α-spin concentration (depletion) of the SOM-ISD and the tropicity of the SO-MICD agree with the (de)­shielding character of the SOC contribution to the ^1^H NMR shift.

**Figure 6:**
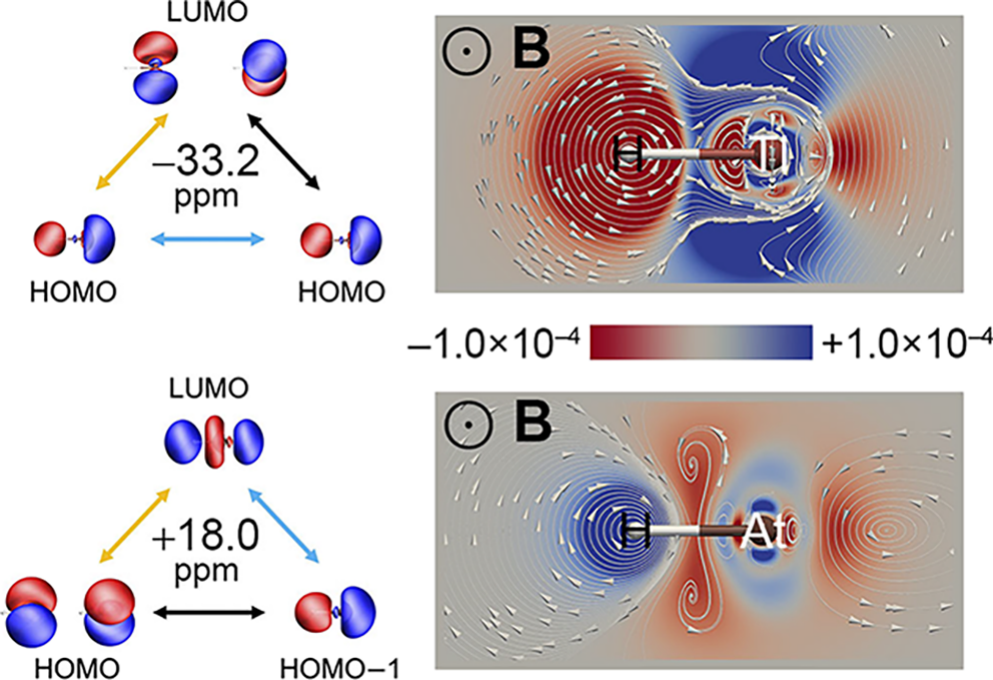
Left: the leading contributions to σ^SO/FC^ of TlH (top) and HAt (bottom) in the framework of PT3 theory. Right: the associated SOM-ISD and SO-MICD sustained by the shown PT3 couplings. The perturbative operators are color-coded as follows: SOC, orange; FC, blue; L, black. The white arrowheads indicate the direction of the SO-MICD.

### The *trans*-Ligand Influence in Linear HMX Systems

#### Molecular Geometry

In the classical Buckingham-Stephens model^28,29^ invoked to explain the characteristic ^1^H NMR shielding of TM hydrido-complexes, the distance between the TM atom and H is considered one of the main parameters that determine the magnitude of the NMR shielding. Therefore, we started our study by focusing on the effect of SOC on the equilibrium geometry of the studied molecules. The optimized M–H bond lengths of molecules **1a**-**1g** and **2a**-**2g** are collected in Table 3. The M–H lengths correlate with the TLI strength of ligand X as expected: the stronger the TLI of X, the longer the *trans* M–H bond. Molecules **1h**-**1j** (**2h**-**2j**) are bonded to neutral ligands and have a different charge state, thereby not following the same trend as **1a**-**1g** (**2a**-**2g**). The interested reader is referred to Supporting Information for more detailed data on these systems. The specific effect of SOC on the molecular geometry is approximated as the distortion of the M–H and M–X bond lengths seen when going from structure optimization at the SR level (with the 1c-ZORA Hamiltonian) to the SO relativistic level (with the 2c-ZORA Hamiltonian). In general, a SO-induced shortening of all distances is observed, regardless of the identity of the central metal atom and the TLI strength of the bonded ligand (see Tables S7 and S8). However, the magnitude of the M–H distance shortening is more pronounced for strong TLI ligands. This can be explained by considering the SO-induced rearrangement in electron density, SO-EDD.^11^ The SO-EDD is a ground state property defined as the difference between electron charge densities with the inclusion and exclusion of SOC.^11,12^ In simple cases where only one heavy atom is present, the SO-EDD is a straightforward means of explaining the effect of SOC on molecular geometry and magnetic response properties in terms of charge reorganization, and also provides a qualitative link between them.^23^ This was previously shown for a series of heavy-atom hydrides including TlH and HAt. In TlH, whose bond length is shortened by SOC in the same way as in **1a**-**1g** (**2a**-**2g**), SO-induced accumulation of electron density in the region between the atoms screens the internuclear repulsion more effectively, and leads to the development of an attractive Hellmann–Feynman force on H. For an analysis of the SO-EDD for compounds **1a**-**1g** along the same lines, see Figure S7. The sign of SO-EDD at the position of the hydrogen atom in H–HA has been shown to correlate with that of the SOM-ISD.^13,23^ Therefore, we continue by analyzing the magnetic response in the following sections.

**Table 3:** M–H Bond Length (in Å), the Total Ligand ^1^H NMR Shielding Constant (σ^H^ in ppm), and the Net MICD Strength Circulating around the Hydrogen Atom (*I* ^H^ in nA T^–1^) Calculated at the SR-4c-DKS and SOR-4c-DKS Levels of Theory for Molecules AuH, **1a**-**1g**, and **2a**-**2g** ^a^ ^,^ ^b^

molecule	X	M–H bond length	σ_SR_^H^	σ_SOR_^H^	Δσ_SO_^H^	*I*_SR_^H^	*I*_SOR_^H^	Δ*I*_SO_^H^
AuH		1.534	+31.0	+51.8	+20.8	+5.96	+7.76	+1.80
**1a**	F	1.562	+34.8	+44.6	+9.8	+6.41	+7.17	+0.76
**1b**	Cl	1.581	+32.3	+36.3	+4.0	+5.65	+5.97	+0.32
**1c**	Ph	1.633	+31.1	+27.0	–4.1	+5.23	+4.83	–0.40
**1d**	CH_3_	1.636	+31.0	+25.9	–5.1	+5.06	+4.57	–0.49
**1e**	H	1.656	+30.0	+21.7	–8.3	+4.85	+4.08	–0.77
**1f**	SiH_3_	1.661	+28.6	+16.0	–12.6	+4.47	+3.33	–1.14
**1g**	BH_2_	1.701	+28.7	+13.2	–15.5	+4.51	+2.72	–1.79
**2a**	F	1.577	+28.5	+30.1	+1.6	+4.63	+4.75	+0.12
**2b**	Cl	1.600	+27.3	+23.2	–4.1	+4.23	+3.85	–0.38
**2c**	Ph	1.635	+27.4	+14.7	–12.7	+4.14	+3.03	–1.11
**2d**	CH_3_	1.641	+27.3	+11.2	–16.1	+3.92	+2.48	–1.44
**2e**	H	1.646	+27.5	+10.8	–16.7	+3.99	+2.48	–1.51
**2f**	SiH_3_	1.665	+26.2	+2.3	–23.9	+3.74	+1.65	–2.09
**2g**	BH_2_	1.669	+27.0	+3.3	–23.7	+3.64	+1.11	–2.53

a For total ligand ^1^H NMR shielding constants and net MICD strengths calculated at the SR-4c and SOR-4c Dirac–Hartree–Fock levels of theory for molecules AuH and **1a**-**1g**, see Table S26.

b For total ligand ^1^H NMR shielding constants and net MICD strengths calculated at the nonrelativistic 4c-DKS level of theory for molecules AuH and **1a**-**1g**, see Table S27.

#### MICD Strengths

The MICD and SO-MICD strengths for molecules **1a**-**1g** (**2a**-**2g**) are collected in Table 3, together with the ligand ^1^H NMR shielding constants for comparison purposes. The analogous SR-MICD strengths, obtained by subtracting the nonrelativistic MICD strengths calculated with an artificial value of the speed of light of 10*c* to the SR-4c-DKS values, are listed for some of the studied molecules in Table S27. AuH, which can be considered as coordinated to a ligand of vanishing TLI, is included as a reference.

The calculated MICD strengths of **1a**-**1g** (**2a**-**2g**) qualitatively agree with the increasing TLI of ligand X, the effects of which are seen in the elongated M–H distances and diminished total NMR shielding constants when going from **1a** (**2a**) to **1g** (**2g**). In short, the inclusion of ligands with strong TLI weakens the MICD strength at both SR and SOR levels of theory. The correlation between MICD strengths and total NMR shielding constants is roughly linear, as shown in Figures S9 and S10; the correlation is stronger for the SOR-4c-DKS values than for the SR-4c-DKS, because the latter are clustered in a small range (or, conversely, SO leads to a larger range of both MICD strengths and NMR shielding constants—cf. the well-known ability of SO-HALA effects to produce ^1^H NMR shifts well outside of the range normally seen in organic chemistry).^9,12,13^ It is also noteworthy that notably smaller MICD strength values are systematically obtained for the mercury­(II) compounds than for those with gold­(I). The relationship between MICD strengths, NMR shieldings, and Au–H bond lengths is examined in more detail in the following subsections. Because of their similar behavior, only the gold­(I) complexes will be discussed below, but the conclusions can be extrapolated to the mercury­(II) systems.

#### MICD Topologies

The inspection of SOC-induced changes in the shape and/or tropicity of the MICD delocalization pathways provides further insight into the role of SOC in the TLI phenomenon. The MICD delocalization pathways for **1a** and **1g** are shown in Figure 7, whereas the decomposition of the MICD and SO-MICD strengths of **1a**-**1g** (**2a**-**2g**) into diatropic and paratropic components can be found in Supporting Information (Tables S22–S25).

**Figure 7:**
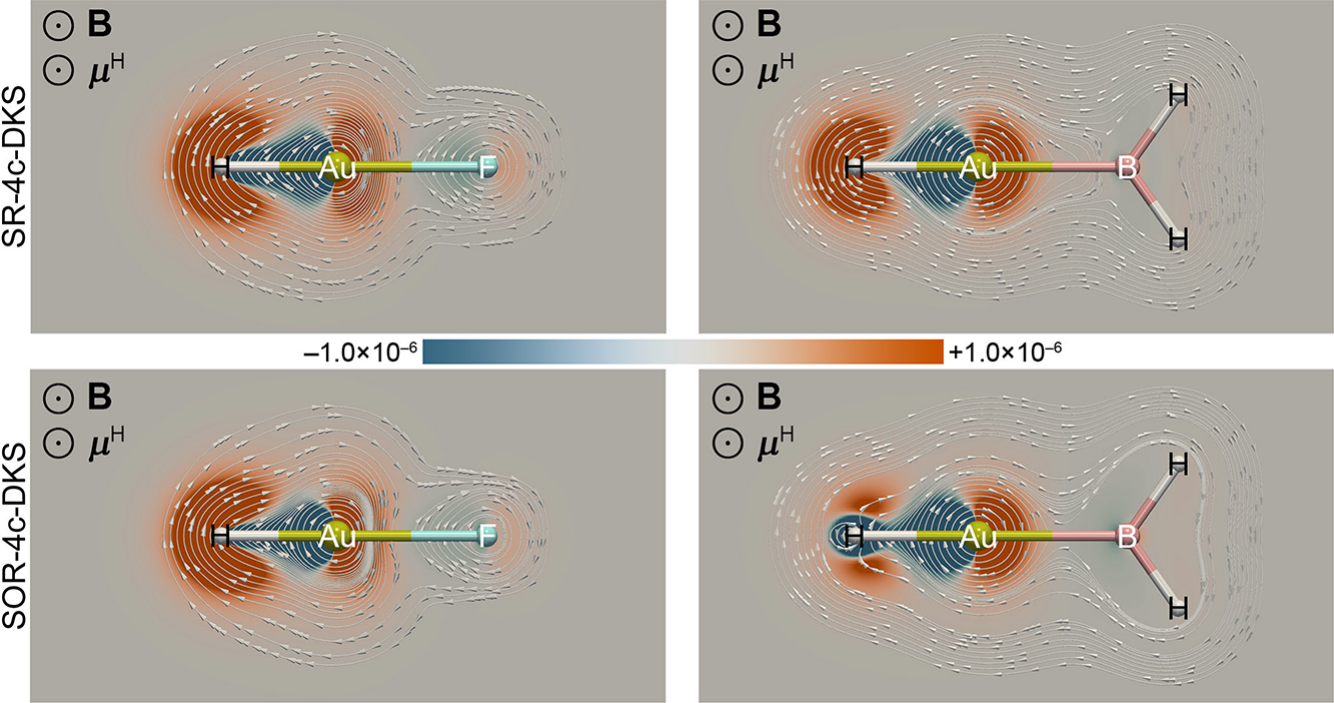
MICD delocalization pathways of molecules **1a** (left) and **1g** (right) calculated at the SR-4c-DKS (top) and SOR-4c-DKS (bottom) levels of theory, superimposed with the positive (in orange) and negative (in blue) contributions to the MSD of H. The white arrowheads indicate the direction of the MICD.

In the case of a weak TLI ligand such as F (Figure 7, left), the inclusion of SOC results in a strengthening of the diatropic current and an increase of the curvature of the MICD toward the Au–H bond. This effect is analogous to that described by Berger et al.^14^ for AuH and results in SO-HALA shielding of H. In contrast, strong TLI ligands weaken the diatropic current. The main effect of this is to push the MICD pathways on the far side of H toward the H nucleus, creating a kink in the pathway rather than (as at SR-4c-DKS level) currents curving smoothly around the H nucleus—see Figure S11; this effect arises from the SO effects overlaying an H-centered paratropic current on the Au-centered diatropic currents. When the TLI is strong enough, as for **1f** (X = SiH_3_) and **1g** (X = BH_2_), this paratropic current locally overrides the diatropic currents, resulting in a paratropic vortex at the H position. This is seen for **1g** in Figure 7, bottom right, which shows the paratropic vortex embedded within the global diatropic current. At the SR level, both **1f** and **1g** do not feature paratropic currents in the surroundings of H. Therefore, the origin of the paratropic vortex is entirely due to SOC. Overall, the SOR-4c-DKS MICD topologies indicate more interatomic delocalization in the H–Au and less in the Au–F regions for **1a**, but the opposite for **1g**.

#### Magnetic Shielding Densities: the Spatial Origin of the SO-HALA NMR (de)­Shielding

Both gold­(I) and mercury­(II) centers sustain diatropic atomic MICDs because of their filled d-shell configuration ([Au^I^] ≡ [Hg^II^]: [Xe] 4f^14^ 5d^10^).^31^ These two series of complexes would therefore be expected to show trends in ligand ^1^H NMR shielding w.r.t. M–H bond length opposite to those proposed by Buckingham and Stephens for ‘true’ TM hydrides, which display paratropic atomic MICDs—i.e. in our systems, shorter M–H bonds would lead to more deshielded (instead of more shielded) H. Indeed, a spectator nucleus (here H) in close vicinity to a diatropic MICD vortex (here M) experiences a deshielding effect, because the directions of the vector potential $\vec{A}$ of its nuclear magnetic moment ${\vec{\mu }}^{H}$ and of the diatropic MICD vortex are parallel in the region that separates them (see eq 7). [As an example, consider the characteristic smaller NMR shieldings of aromatic protons w.r.t. nonaromatic analogues induced by the net diatropic MICD sustained by aromatic rings, e.g. benzene (Figure 1).^6,37,67^ ] However, it should also be considered that the directions of the magnetic vector potential and of the vortex are opposite in the far half of the latter (i.e., on the other side of the metal atom), compensating for or even entirely counteracting the deshielding effects.

We show in Figure 7 the spatial distribution of the out-of-plane component of the magnetic shielding density (MSD) of **1a** and **1g** calculated at the SR-4c-DKS and SOR-4c-DKS levels of theory, superimposed with the direction of the MICD. Whether the current has shielding or deshielding contributions on H depends on whether its direction appears clockwise or anticlockwise relative to H (formally, whether the dot product of the current $\vec{j}$ with the negative of the vector potential $\vec{A}$ in eq 7 is positive or negative). So, looking at the SR-4c-DKS currents (Figure 7, top) we see shielding effects (colored orange) where the global diatropic (clockwise) currents pass around H to its left, and to the right of Au, where the local diatropic currents are also clockwise relative to H. However, along the H–Au bond, the local diatropic currents around Au are moving in an overall anticlockwise direction relative to H (having an upward component), and are therefore deshielding (colored blue). The partition of the total ^1^H NMR shielding constant into atomic contributions at the all-electron exact two-component (sc-X2C) level of theory obtained with GIMIC is presented in Table 4. The sc-X2C level of theory should be considered a good approximation to the SR-4c-DKS one. The positive contribution of Au is roughly two times larger than the negative one, resulting in an overall shielding contribution of Au to H. The modification of the topology of the MICD vector field induced by strong TLI ligands together with the slight increase of the Au–H distance leaves H less exposed to the shielding part of the Au contributions, and consequently reduces the positive value while keeping the negative one almost constant—compare the relative shielding induced by Au of **1a** (39%) to that of **1g** (27%). As a result, the NMR shielding constant of H diminishes upon increasing the TLI of ligand X. Then, at least at the SR level, the Buckingham-Stephens model explains the interplay between TLI and NMR shielding constants as a function of M–H bond lengths. However, the picture is different when SOC effects are included. The increased curvature of the MICD pathways of **1a** around the Au–H bond reduces the extent of the deshielding region between H and Au, leading to increased shielding of H. For **1g**, the formation of a paratropic vortex around H is the main source of deshielding. The specific changes in the strength and shape of the MICD induced by the variable TLI of the *trans* ligands, rather than the slight changes in the M–H distance, are the determining factors for the total (de)­shielding effect on the spectator nucleus. The difference between the SOR and SR magnetic shielding densities in Figure S12 demonstrates that the changes most relevant for the total NMR shielding take place in the surroundings of H, whereas those originating around the metal itself are marginal.

**Table 4:** Atomic Contributions to the Total ^1^H NMR Shielding Constant for Molecules **1a** and **1g**, Calculated at the sc-X2C-RI-DFT/PBE0/x2c-QZVPall-s Level of Theory (All Values Are Given in ppm)

		molecule	
		**1a**	**1g**
total	total	36.73 (100)	29.25 (100)
	positive	53.93	43.83
	negative	–17.20	–14.58
H	total (%)	21.90 (60)	20.88 (71)
	positive (%)	26.29	21.89
	negative (%)	–4.39	–1.01
Au	total (%)	14.26 (39)	7.97 (27)
	positive (%)	26.76	20.89
	negative (%)	–12.50	–12.92
X	total (%)	0.57 (<1)	0.40 (<1)
	positive (%)	0.87	1.05
	negative (%)	–0.30	–0.65

The topology of the MICD at the SOR-4c-DKS level may provide insight into the character of the M–H bond and predict its reactivity. In **1a**, the Au–H bond and the fluoride ligand sustain independent local current vortices, which in turn are embedded in the global current. This may suggest a significant degree of covalency of the Au–H bond, and a preferred reactivity toward homolytic splitting. Whereas the H–Au bond seems to form a molecular unit separated from the fluoride ligand in **1a**, both the H–Au and Au–X regions seem to be more balanced in **1g**. The formation of a vortex on H in **1g** suggests loss of covalency and more pronounced reactivity as a true hydride. The connection between the tropicity of SO-MICD and the sign of SOM-ISD for molecules **1a** and **1f** is also in very good agreement, see Figure 8. This is because the SO effects in the studied systems are predominantly linear, see also ref 14.

**Figure 8:**
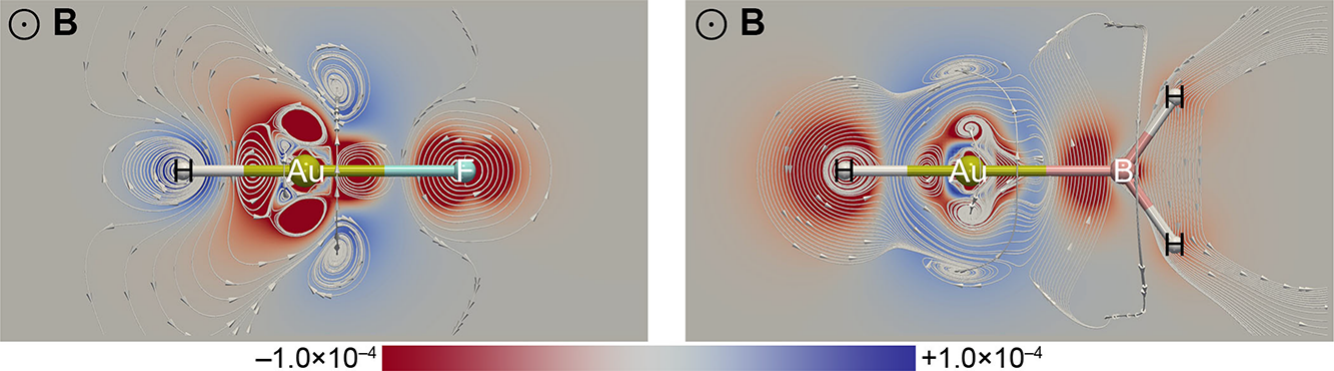
Out-of-plane component of the SOM-ISD, superimposed with the direction of the SO-MICD for molecules **1a** (left) and **1g** (right). The white arrowheads indicate the direction of the SO-MICD.

#### Partition of TLI into Structural and Electronic Components

Because the M–H distance is a key parameter in the classical explanation of the dependence of the ^1^H NMR shifts on the TLI,^28,29^ we have calculated the MICD strength as a function of the Au–H distance for AuH, see Figure 9. The advantage of calculating this property for AuH is that, because it does not possess a ligand *trans* to H, the results provide an unobstructed view of structural *trans*-ligand effects. In a sense, this artificial model can be understood as the Buckingham-Stephens limit for gold­(I) complexes, where all variations of the ^1^H NMR shifts depend only on the Au–H distance. To demonstrate this effect we have varied the bond length from 1.0 to 1.8 Å in steps of 0.1 Å. The values at longer distances are not reliable due to a non-negligible multiconfigurational character of the wave function. The net MICD of AuH is completely diatropic without any paratropic current for all distances. As can be seen in Figure 9, the dependence of the MICD strength upon the Au–H bond length is nonlinear,^13^ with a steeper curve at distances shorter than the calculated equilibrium distance of 1.53 Å, and a flatter one at longer distances. The values obtained at the SOR-4c-DKS (blue crosses in Figure 9) level of theory are consistently higher than those at the SR-4c-DKS (red crosses) level, although the difference due to SOC (black crosses) is not constant over the considered distance interval. The contribution of SOC to the total MICD strength increases with the Au–H distance in a manner similar to that observed for SO-HALA NMR shifts, see Figure 9 in ref 13 and Figure S13 in Supporting Information. When the MICD strength is expressed as a function of the associated NMR shielding constant, a straight line is obtained for both levels of theory, see Figure S14. This is because the streamlines measured by plane integration of the MICD are those involved in the intense shielding contributions to the left of H in Figure 7, and the streamlines not measured (along the Au–H bond and on the other side of Au) are a mixture of shielding and deshielding – i.e. there is significant cancellation in the effects not measured by MICD strength. This is also reflected in the atomic contributions in Table 4.

**Figure 9:**
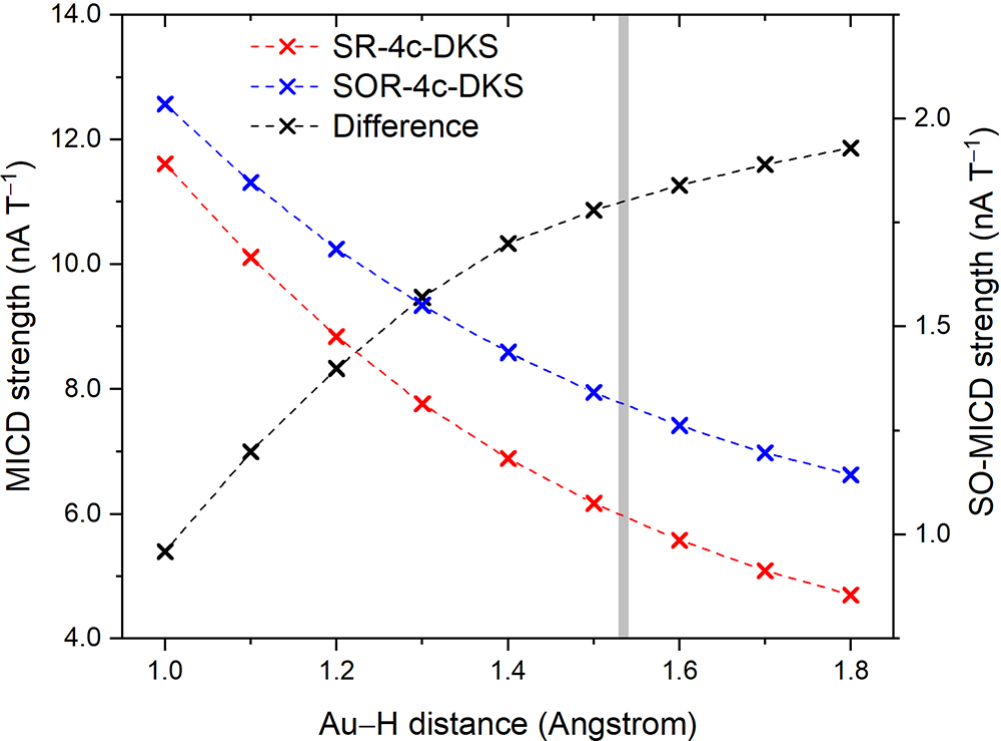
MICD strength at the SR-4c-DKS (red crosses) and SOR-4c-DKS (blue crosses) levels of theory and the derived SO-MICD strength (black crosses) as a function of the Au–H distance for AuH. The gray bar indicates the optimized equilibrium distance of AuH.

We can further analyze the TLI on the MICD strength by conceiving of the **HMX** complexes **1a**-**1g** as built by taking an AuH molecule and stretching the Au–H bond, then adding a ligand. For each complex, we then define the structural value [*I* ^H^(structural)] as that calculated for the stretched AuH subunit in isolation; and the electronic effects [*I* ^H^(electronic)] as the change made by then adding the X ligand, i.e. the difference between the values for **HMX** [*I* ^H^(total)] and AuH at the same Au–H bond length [*I* ^H^(structural)],

$$I^{H}\left(\mathrm{electronic}\right)=I^{H}\left(\mathrm{total}\right)-I^{H}\left(\mathrm{structural}\right)$$ 8

The structural effect of the SOC-induced contraction of the Au–H bond lengths on the MICD strengths is negligible, as it is of the order of tenths of pm, see Tables S7 and S8. The deviation of the calculated MICD strengths of molecules **1a**-**1g** from the curve for AuH in Figure 10 demonstrates that the electronic structure of the molecules does indeed play a role, the importance of which varies dramatically depending on whether SOC effects are considered or not. Whereas at the SR level (in red) the values are somewhat clustered around the line of AuH calculated at the same level of theory, at the SOR level the values depart considerably (in blue). When SOC is considered, the coordination of ligands reduces the MICD strength of AuH regardless of their TLI strength (all blue dots are below the blue dashed line in Figure 10). Thus, even coordination of a weak TLI ligand, such as F (**1a**), reduces the MICD strength compared to that of AuH.

**Figure 10:**
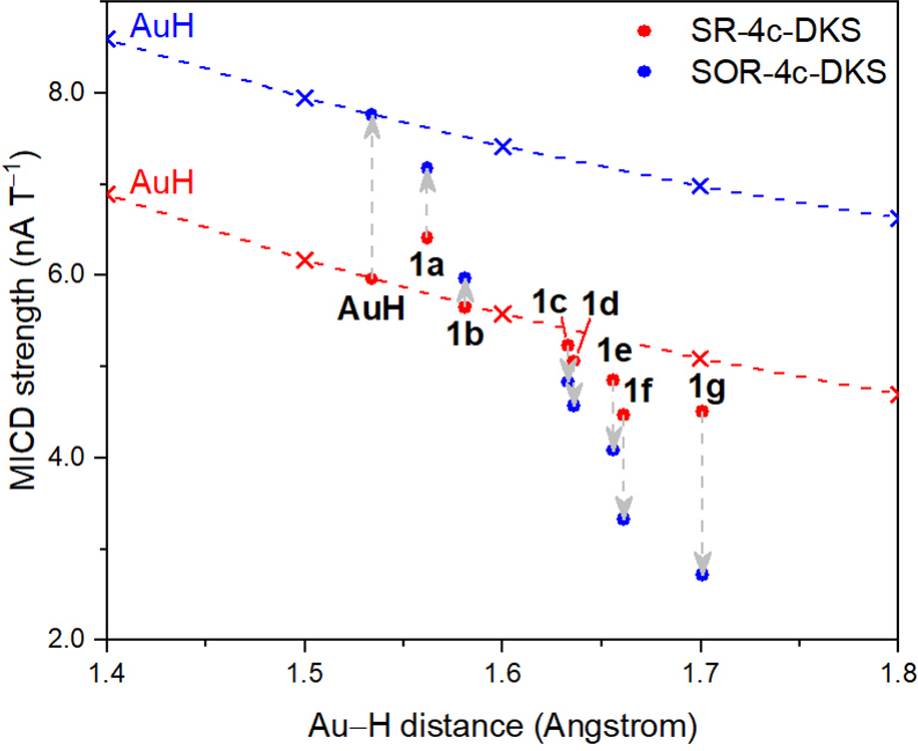
MICD strength as a function of the Au–H distance for molecules AuH [crosses, *I* ^H^(structural)] and **1a**-**1g** [points, *I* ^H^(total)] calculated at the SR-4c-DKS (*I*_SR_^H^, in red) and SOR-4c-DKS (*I*_SOR_^H^, in blue) levels of theory. The gray dashed arrows indicate the SO-induced departure of the MICD strengths of **1a**-**1g** [Δ*I*_SO_^H^(total)]. For values, see Tables S28 and S29.

From the application of eq 8 to the SOR-4c-DKS and SR-4c-DKS levels and taking the difference (Δ*I*_SO_^H^ = *I*_SOR_^H^ – *I*_SR_^H^) it follows that

$$\Delta I_{\mathrm{SO}}^{H}\left(\mathrm{electronic}\right)=\Delta I_{\mathrm{SO}}^{H}\left(\mathrm{total}\right)-\Delta I_{\mathrm{SO}}^{H}\left(\mathrm{structural}\right)$$ 9

The SO-MICD as a function of the Au–H distance is shown in Figure 11. Even though the elongation of the Au–H distance affects the strength of the SO-MICD regardless of its origin, the sign and magnitude of this SOC correction depends heavily on whether the elongation is due to structural [Δ*I*_SO_^H^(structural), crosses] or electronic [Δ*I*_SO_^H^(electronic), the length of the dashed arrows] TLI. This demonstrates the serious limitations of the classical nonrelativistic Buckingham-Stephens model, and the necessity of including relativistic effects for a proper description of ligand NMR of heavy TM complexes.

**Figure 11:**
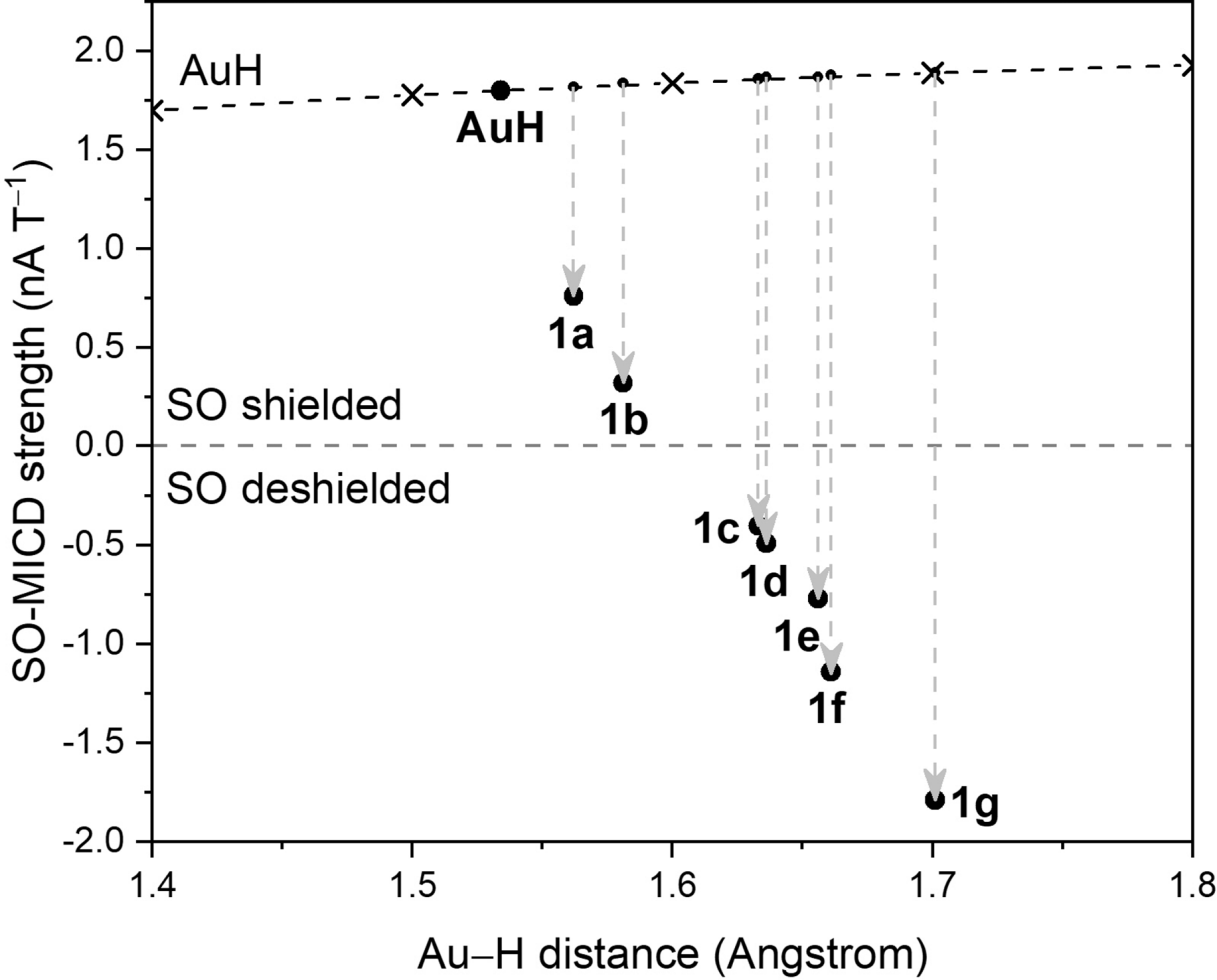
SO-MICD strength as a function of the Au–H distance for molecules AuH [Δ*I*_SO_^H^(structural), crosses] and **1a**-**1g** [Δ*I*_SO_^H^(total), points]. The gray dashed arrows indicate the deviation of the SO-MICD strengths of molecules **1a**-**1g** from the values of AuH at the same Au–H distance as the **HMX** complex’s equilibrium bond length [Δ*I*_SO_^H^(electronic)]. For tabulated values, see Table S29.

The TLI of a ligand X on the electronic structure of **HMX** can also be evaluated by scanning the MICD strength as a function of the Au–X bond length.^32,33^ Again, we consider systems **1a** (X = F) and **1g** (X = BH_2_) as representative examples of weak and strong TLI, respectively. The plots of the net MICD strength as a function of the Au–H and Au–X bond lengths can be found in Figures S17 and S18. For **1a**, the effect of distorting the Au–H bond from its equilibrium distance is, for all Au–F bond lengths considered, very similar to that observed for AuH in Figure 9. This may be due to the low covalency of the Au–F bond, since F is a very weak TLI ligand, resulting in the overall MICD strength being dominated by the behavior of the Au–H moiety. The picture of **1g** is rather different. Here, both the elongation of the Au–H bond and the compression of the Au–B bond from their respective equilibrium lengths give rise to a paratropic vortex on H strong enough, for the shortest Au–B lengths, to entirely counteract the effects of the diatropic currents, leading to an overall negative net MICD strength. It is evident here that a correct description of the TLI can only be achieved at a level of theory that provides a good treatment of AuH.

#### Molecular Orbital Contributions to NMR Shieldings and MICDs

We note that the effect of SO interaction on the ^1^H NMR shielding decreases from **1a** to **1g**, i.e. with the substituent order F > Cl > Ph > CH_3_ > H > SiH_3_ > BH_2_. This is almost, but not quite, the trend in Pauling electronegativity (χ_P_) of the atom bonded to gold, χ_P_(F) = 3.98 > χ_P_(Cl) = 3.16 > χ_P_(C) = 2.55 > χ_P_(H) = 2.20 > χ_P_(B) = 2.04 > χ_P_(Si) = 1.90.^68^ We therefore frame our explanation in terms of two effects: the general trend matches χ_P_, but there is an additional deshielding enhancement for **1g** with X = BH_2_.

The larger number of NMR-active MOs and the notably smaller size of their contributions to σ^SO/FC^ as compared to TlH and HAt (vide supra) makes the interpretation of the PT3 analysis for molecules **1a**-**1g** less straightforward. Thus, to approach it in a systematic manner, we start by contemplating the ligandless molecule AuH,^13^ then extending the analysis to the symmetric molecule **1e** with X = H, and finally observing the effect of replacing X by more (**1a**, X = F) or less (**1f**, X = SiH_3_) electronegative moieties. There are several PT3 couplings contributing to either hydride shielding or deshielding, but they can be easily grouped according to the symmetry and AO composition of the involved MOs. Assuming that the principal axis of the molecule is collinear with the *z* axis, the most relevant MOs include the σ-bonding combination of the 1s AO of H and the 5d_z^2^_ AO of Au (σ_sd_), the σ-bonding combination of the 1s AO of H and the 6s AO of Au (σ_ss_), and the σ-bonding combination of the 1s AO of H and the 6p_*z*_ AO of Au (σ_sp_). A cartoon representation of these MOs for AuH and **1e** is shown in the energy diagram of Figure 12, and their Mulliken population analysis can be found in Table S30. The PT3 shielding mechanism of AuH has already been discussed in detail in ref 13 but, for the convenience of the reader, we recall that it originates in the coupling between the occupied bonding σ_sd_ MO and nonbonding 5d_*xz*_/5d_*yz*_ AOs of Au, with the vacant bonding σ_sp_ MO, as shown in Figure 12. The coordination of H as the *trans* ligand in molecule **1e** has significant effects on the character and relative energies of the MOs. First, the presence of another 1s AO expands the two-centered σ MOs into three-centered σ MOs of *gerade* (σ_sd_, σ_ss_) or *ungerade* (σ_sp_) parity; and second, the formerly vacant σ_sp_ is now occupied and becomes HOMO–1. This last point is vital for the magnetic response of **1e**, since when this MO is occupied, it allows for a very efficient deshielding coupling (−5.1 ppm) that cannot arise in AuH, and the shielding coupling described for AuH is no longer possible. The dominant PT3 coupling is shown in Figure 12 involving σ_sp_ twice and the vacant 6p_*x*_/6p_*y*_ AOs on Au. The tropicity of the SO-MICD sustained by the coupled MOs, and the sign of the SOM-ISD in the surroundings of H, both agree with the sign of σ^SO/FC^, similarly to TlH and AtH.

**Figure 12:**
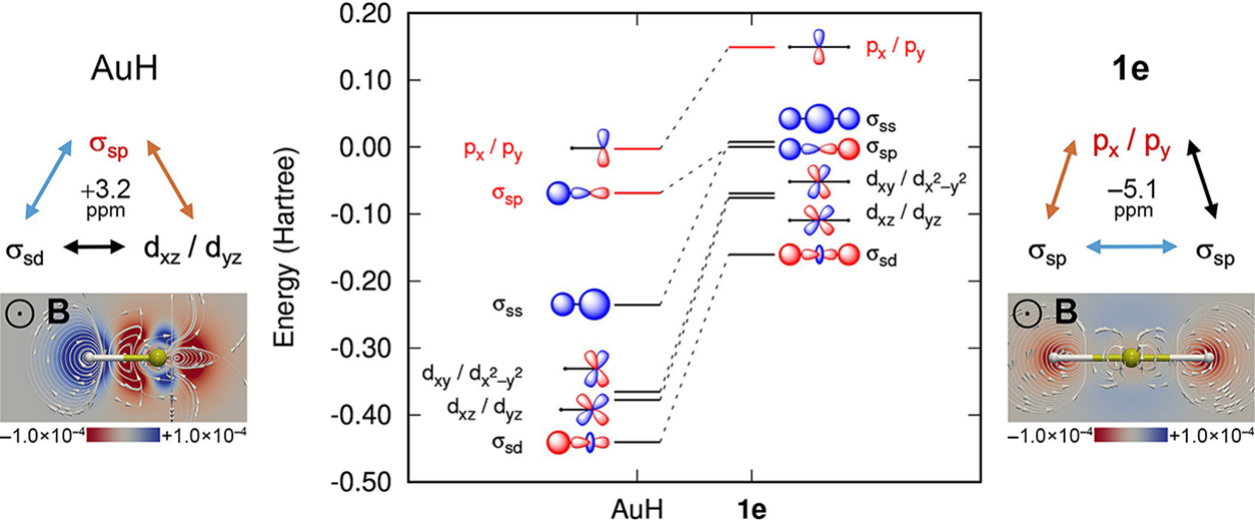
Center: schematic energy diagram for AuH (left) and **1e** (right) with the labeling of the most relevant MOs. Vacant orbitals are color-coded in red. Left and right: the leading contribution to σ^SO/FC^ for AuH (left) and **1e** (right) in the framework of PT3 theory. The perturbative operators are color-coded as follows: SOC, orange; FC, blue; L, black. The associated SOM-ISD and SO-MICD sustained by the PT3 couplings appear below them. The white arrowheads indicate the direction of the SO-MICD.

The replacement of one H with another ligand X of greater or lower electronegativity than H will, respectively, decrease or increase the H-character of the σ orbitals, thus decreasing or increasing their effect on properties around H. We will focus on molecules **1a** with X = F and **1f** with X = SiH_3_, which show respectively the weakest and the strongest electronic TLI effects of the series.

(In the discussion that follows, please note that significant involvement of ligand p-orbitals in the perturbations complicates the analysis, causing exceptions to some of the patterns noted in refs 11 and 12.)

As in **1e**, the largest deshielding originates from the σ_sp_ ↔ π_pp_*↔ σ_sp_ coupling. Note that the 6p_*x*_/6p_*y*_ Au AOs are here replaced by doubly-degenerate MOs featuring small antibonding contributions from p AOs on X (π_pp_*). The efficiency of such coupling varies along the TLI series from **1a** (−0.7 ppm) to **1e** (−5.1 ppm) and **1f** (−7.7 ppm). The reason for this increase is the reduction of the energy gap between σ_sp_ and π_pp_*, as a consequence of the destabilization of σ_sp_ induced by the TLI. This deshielding effect is somewhat diminished by shielding couplings involving π-bonding combinations of 5d_*xz*_/5d_*yz*_ orbitals on Au and p_*x*_/p_*y*_ orbitals on X (π_dp_). These π_dp_ MOs play a similar role in here to that of 5d_*xz*_/5d_*yz*_ Au AOs in the shielding coupling of AuH. This is the dominant mechanism for the SO/FC shielding of molecule **1a** (+1.4 ppm).

As we have mentioned already, the case of molecule **1g** with X = BH_2_ is a special case, since it displays the largest SOC effect among the **1a**-**1g** series despite not being bonded to the least electronegative ligand. This is due to two reasons: first, the high anisotropy of the BH_2_ ligand breaks the degeneracy of the π_dp_/π_dp_* and π_pp_* MOs; and second, the presence of an empty 2p orbital on B perpendicular to the molecular plane raises the energy of the out-of-plane π_dp_* MOs, which now becomes the LUMO. The presence of this low-lying LUMO enables new deshielding σ_sp_ ↔ π_dp_*↔ σ_ss_ couplings that cannot be established in any of the other molecules. The energy levels of the molecules **1a**, **1f**, and **1g** with labeling of the MOs are shown in Figure 13.

**Figure 13:**
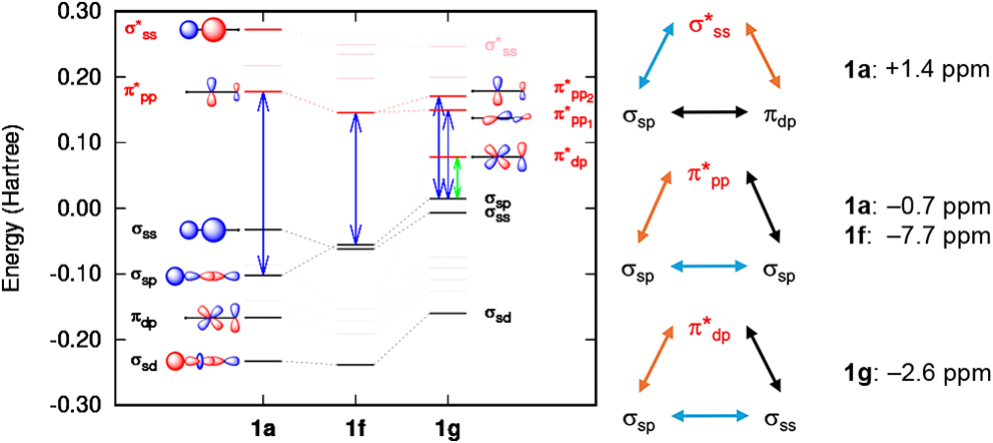
Left: schematic energy diagram for **1a**, **1f**, and **1g** with labeling of the most relevant MOs. The blue arrows highlight the reduction of the energy gap between σ_sp_ and π_pp_*, whereas the green arrow indicates the coupling between σ_sp_ and π_dp_* for **1g**. Vacant orbitals are color-coded in red. Right: the leading contributions to σ^SO/FC^ for molecules **1f** (top), **1a** (center), and **1g** (bottom) in the framework of PT3 theory. The perturbative operators are color-coded as follows: SOC, orange; FC, blue; L, black.

## Summary and Perspectives

We have presented an analysis that makes complementary use of NMR shielding constants and magnetically induced current densities (MICDs) in characterizing bonding between heavy and light atoms. Whereas NMR shielding probes the HA–LA effect at the position of the light atom and can be experimentally measured, MICDs give full spatial information on the response of the electron density to an applied magnetic field, but cannot be measured in practice (although currents are, theoretically, an observable). However, the total induced current is the source of the NMR shielding at any position in the molecule, including those of the atomic nuclei in the system. We have also demonstrated the effect of spin–orbit coupling on the MICDs and how this relates to the magnetic response observed as the NMR shielding. This was first shown in diatomic HA–LA molecules and was extended to linear **HMX** molecules containing Au^I^ and Hg^II^ metal centers (M) and ligands (X) of varying *trans*-ligand influence (TLI). A systematic study of the relation of MICD strength to bond length has allowed for an effective separation into direct and indirect TLI contributions, i.e. into effects on the electronic structure and the geometry, respectively. Our results demonstrate that the well-established off-center ring current model by Buckingham and Stephens^28,29^ is an oversimplification of a more complex mechanism involving SOC effects on the shape of the MICD and NMR shielding. Clearly, a complete understanding of NMR shielding of ligand atoms in transition-metal complexes requires accurate electronic structure calculations and innovative analyses of the magnetic response. This understanding is crucial because these effects also affect the reactivity of the complexes,^69–71^ including H-transfer processes.

## Supplementary Material



## Data Availability

The computational data are available in the ioChem-BD repository^72^ and can be accessed via https://doi.org/10.19061/iochem-bd-6-656.

## Notes

MO analyses, diamagnetic and paramagnetic NMR shielding constants, partition of MICD strengths into diatropic and paratropic components, and calculated data for compounds **1h-1j** and **2h-2j** (PDF)
