# Supplementary material for: Link between Spin–Orbit Relativity and Magnetically Induced Current Densities in Heavy-Atom Hydrides: trans-Ligand Influence
Source: JACS Au. 2026 Jun 2;6(6):3323–35. doi: 10.1021/jacsau.6c00346 (PMC13291875; doi:10.1021/jacsau.6c00346)
Supplement: Supplementary file 1 [file au6c00346_si_001.pdf]

Supporting Information

Link between Spin–Orbit Relativity and Magnetically Induced Current Densities in Heavy-Atom Hydrides: *trans*-Ligand Influence

Daniel Blasco,[a] Jan Novotný,[ab] James R. Asher,[c] Raphael J. F. Berger,[d] Stanislav Komorovsky,*[c] and Radek Marek*[ab]

[a] CEITEC – Central European Institute of Technology, Masaryk University, 62500 Brno, Czechia
[b] Department of Chemistry, Faculty of Science, Masaryk University, 62500 Brno, Czechia
[c] Institute of Inorganic Chemistry, Slovak Academy of Sciences, 84536 Bratislava, Slovakia
[d] Department of Chemistry and Physics of Materials, Paris Lodron University of Salzburg, 5020 Salzburg, Austria

Email: stanislav.komorovsky@savba.sk; radek.marek@ceitec.muni.cz

Table S1: The calculated SO contribution to the bond dissociation energies [Δ(BDE) in kcal mol^{-1}] of TlH, HAt, and AuH, considering heterolytic bond splitting patterns

Molecule	Δ(BDE) ($H^+ + X^-$)	Δ(BDE) ($H^- + X^+$)
TlH	+17.47	−4.16
HAt	−1.04	+35.10
AuH	−0.48	−1.31

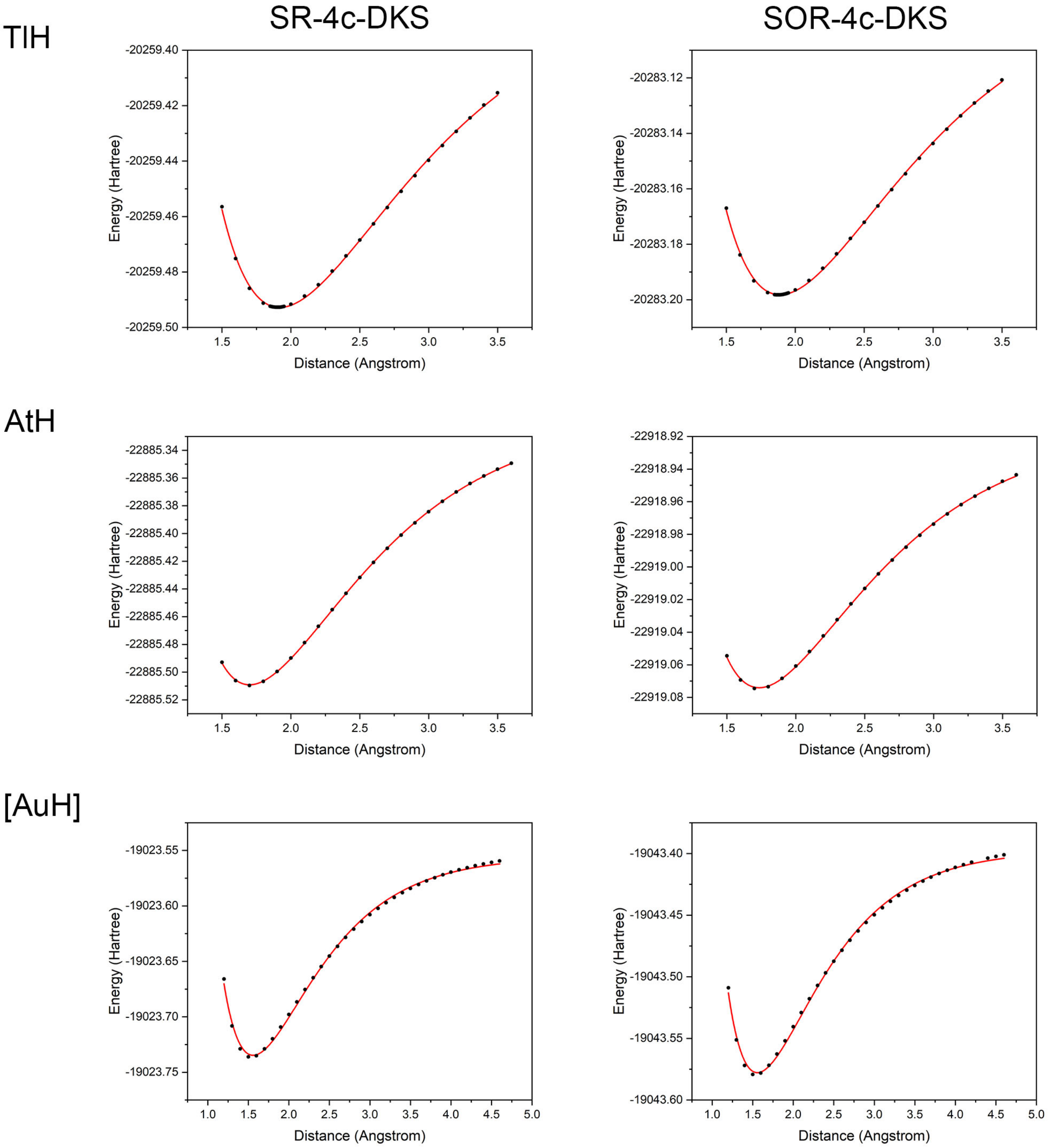

Figure S1: The total electronic energy of molecules TlH (top), HAt (middle), and AuH (bottom), calculated at the SR-4c-DKS (left) and SOR-4c-DKS (right) levels of theory as a function of the internuclear distance. The fit to Equation 3 is shown as a red line.

Table S2: The fitting parameters of the total electronic energy of molecules TlH, HAt, and AuH, calculated at the SR-4c-DKS and SOR-4c-DKS levels of theory, as a function of the internuclear distance

Level of theory	r_e (Å)	a	D_e (kcal mol^{-1})	k_e (N m^{-1})	Adj. R^2
TlH					
SR-4c-DKS	1.91934 ± 0.00197	1.04330 ± 0.01033	51.1 ± 0.6	111 ± 3	0.99965
SOR-4c-DKS	1.88995 ± 0.00162	1.06716 ± 0.00884	49.7 ± 0.4	113 ± 2	0.99977
HAt					
SR-4c-DKS	1.70047 ± 0.00112	1.24828 ± 0.00527	84.7 ± 0.2	264 ± 2	0.99997
SOR-4c-DKS	1.73438 ± 0.00176	1.26091 ± 0.00976	69.4 ± 0.3	220 ± 4	0.99988
AuH					
SR-4c-DKS	1.55996 ± 0.00323	1.30431 ± 0.01439	78.2 ± 0.5	266 ± 6	0.99890
SOR-4c-DKS	1.55864 ± 0.00319	1.30745 ± 0.01444	78.8 ± 0.5	269 ± 6	0.99893

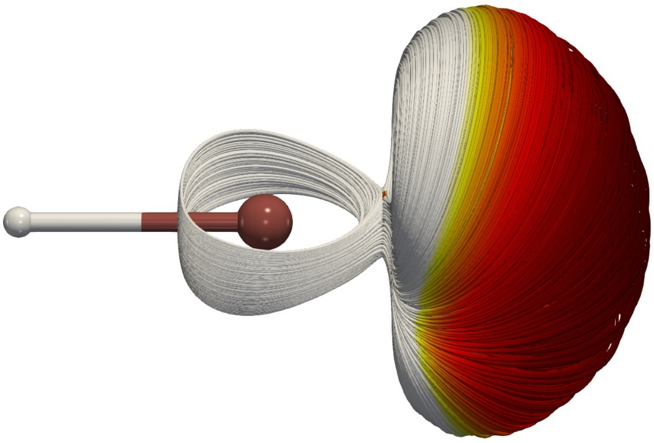

Figure S2: A detail of the MICD delocalization pathways of TlH showing the poloidal currents around Tl. The pathways are color-coded according to their strength using the color scale white ($1 \cdot 10^{-1}$ nA T $^{-1}$)-yellow-orange-red-black ($1 \cdot 10^{-6}$ nA T $^{-1}$).

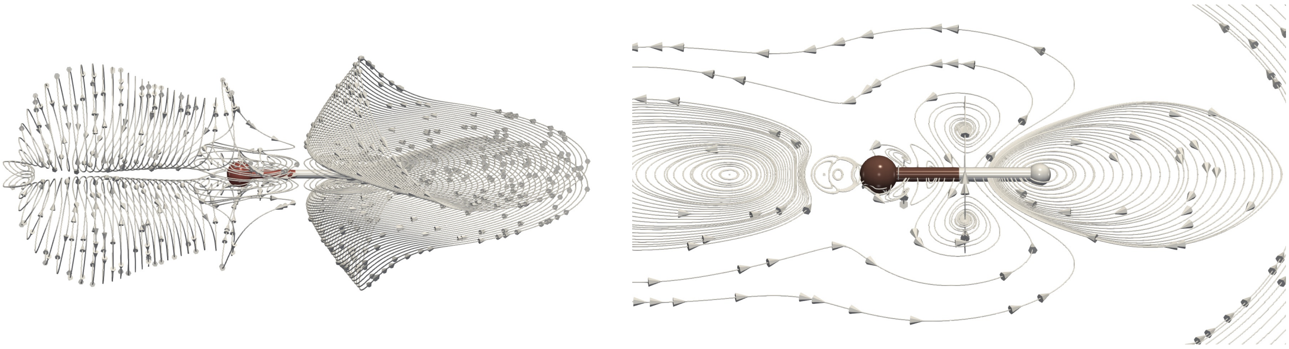

Figure S3: The SO-MICD delocalization pathways of TlH (left) and HAt (right). The arrowheads indicate the direction of the SO-MICD. The external magnetic field vector is perpendicular to the depicted molecular plane. Color code: H, white; At, dark brown; Tl, light brown.

Table S3: The energy (in Hartree) and the atomic orbital composition (in %) of the non-relativistic molecular orbitals involved in the leading contributions to $\sigma^{\mathrm{FC/SO}}$ of molecules TlH and HAt in the framework of PT3 theory

Molecule		MO	E	HA AO				H AO
				6s	6p$_x$	6p$_y$	6p$_z$	1s
TlH	41	HOMO	−0.17019	26	0	0	37	36
	42	LUMO	−0.09208	0	14	86	0	0
	43	LUMO	−0.09208	0	86	14	0	0
	44	LUMO+1	0.02760	3	0	0	70	24
HAt	41	HOMO−1	−0.40639	13	0	0	53	33
	42	HOMO	−0.27949	0	60	40	0	0
	43	HOMO	−0.27949	0	40	60	0	0
	44	LUMO	−0.03437	1	0	0	42	50

Table S4: The energy (in Hartree) and the atomic orbital composition (in %) of the fully-relativistic molecular spin-orbitals involved in the leading contributions to $\sigma^{\mathrm{FC/SO}}$ of molecules TlH and HAt in the framework of PT3 theory

Molecule		MO	E	HA AO				H AO
				6s	6p$_x$	6p$_y$	6p$_z$	1s
TlH	41	HOMO	−0.21294	11 (α, Re)	1 (β, Re)	1 (β, Im)	36 (α, Re)	49 (α, Re)
	42	LUMO	−0.07067	0	48 (α, Re)	48 (α, Im)	0	3 (β, Re)
	43	LUMO	−0.04872	0	50 (α, Re)	50 (α, Im)	0	0
	44	LUMO+1	0.02028	0	0	0	59 (α, Re)	29 (α, Re)
HAt	41	HOMO−1	−0.42556	3 (α, Re)	7 (β, Re)	7 (β, Im)	49 (α, Re)	32 (α, Re)
	42	HOMO	−0.30803	0	41 (α, Re)	41 (α, Im)	3 (β, Re)	14 (β, Re)
	43	HOMO	−0.24543	0	50 (α, Re)	50 (α, Im)	0	0
	44	LUMO	−0.04212	0	2 (β, Re)	2 (β, Im)	39 (α, Re)	56 (α, Re)

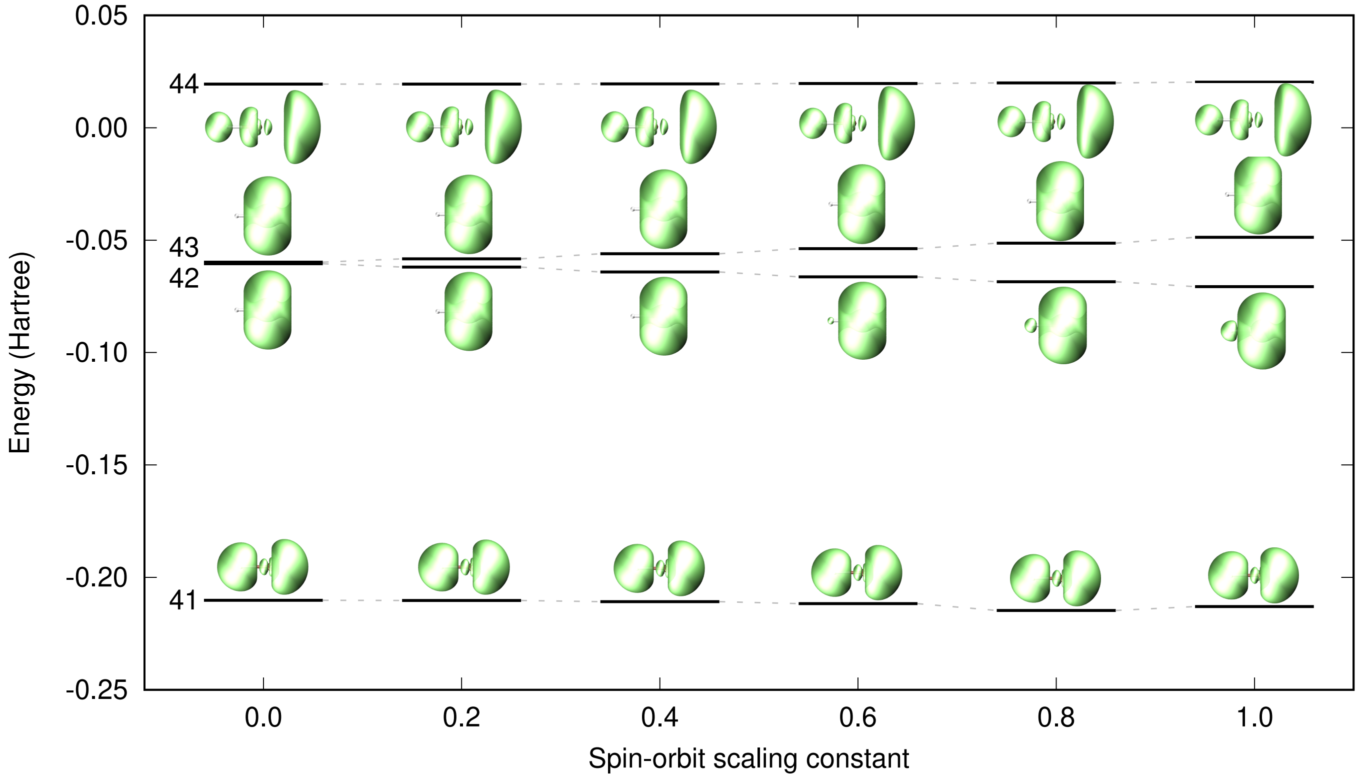

Figure S4: The frontier molecular orbitals of TlH upon increments of the spin-orbit scaling constant from 0.0 (SR-4c-DKS) to 1.0 (SOR-4c-DKS) in steps of 0.2.

Table S5: The net MICD strength circulating around the hydrogen atom and its partition into diatropic and paratropic components for the most relevant PT3 coupling of TlH (all quantities are given in nA T^{-1})

	Spin-orbit scaling constant					
	0.0	0.2	0.4	0.6	0.8	1.0
I^{H} (total)						
Net	+1.54	−0.56	−3.14	−6.28	−10.05	−13.56
Diatropic	+1.97	+0.69	+0.34	+0.35	+0.44	+0.43
Paratropic	−0.43	−1.25	−3.48	−6.63	−10.49	−13.99
I^{H} (PT3 coupling)						
Net	+0.18	−0.93	−2.34	−4.15	−6.40	−9.13
Diatropic	+1.11	+0.56	+0.22	+0.12	+0.14	+0.18
Paratropic	−0.94	−1.49	−2.56	−4.26	−6.53	−9.31

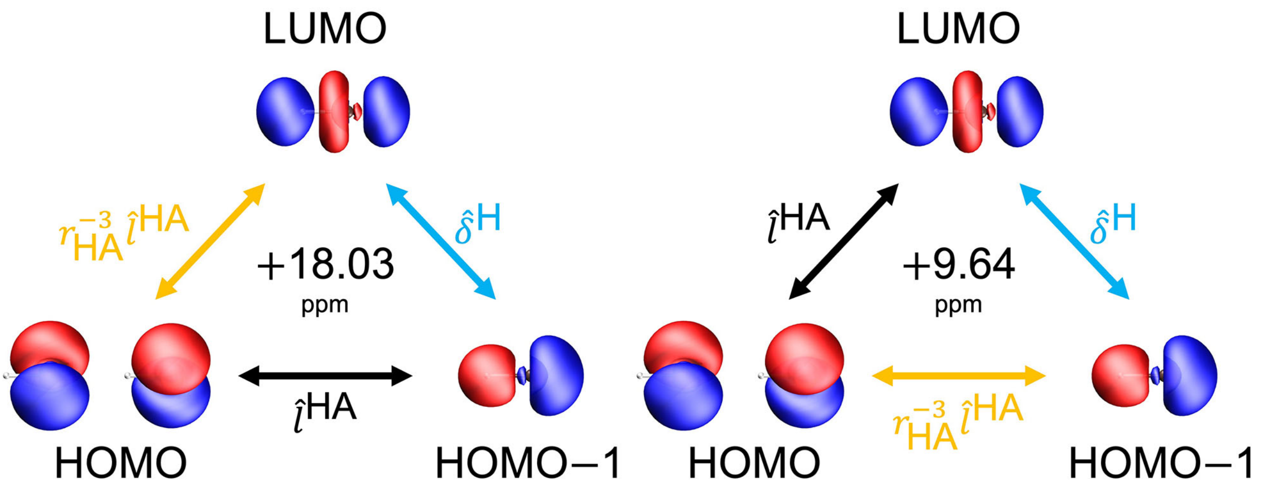

Figure S5: The leading shielding contributions to $\sigma^{SO/FC}$ of HAt in the framework of PT3 theory, where $r_{HA}^{-3}\hat{l}^{HA}$ is the SOC operator, $\hat{l}^{HA}$ is the external magnetic field–angular momentum coupling operator, and $\hat{\delta}^{H}$ is the Fermi contact operator.

Table S6: The net MICD strength circulating around the hydrogen atom and its partition into diatropic and paratropic components for the most relevant PT3 coupling of HAt (all quantities are given in nA T^{-1})

	Spin-orbit scaling constant					
	0.0	0.2	0.4	0.6	0.8	1.0
I^{H} (total)						
Net	+5.91	+6.93	+7.78	+8.46	+9.01	+9.23
Diatropic	+5.91	+6.93	+7.78	+8.46	+9.01	+9.23
Paratropic	−0.00	−0.00	−0.00	−0.00	−0.00	−0.00
I^{H} (PT3 coupling)						
Net	+4.58	+5.25	+5.79	+6.23	+6.58	+6.88
Diatropic	+4.58	+5.25	+5.79	+6.23	+6.58	+6.88
Paratropic	−0.00	−0.00	−0.00	−0.00	−0.00	−0.00

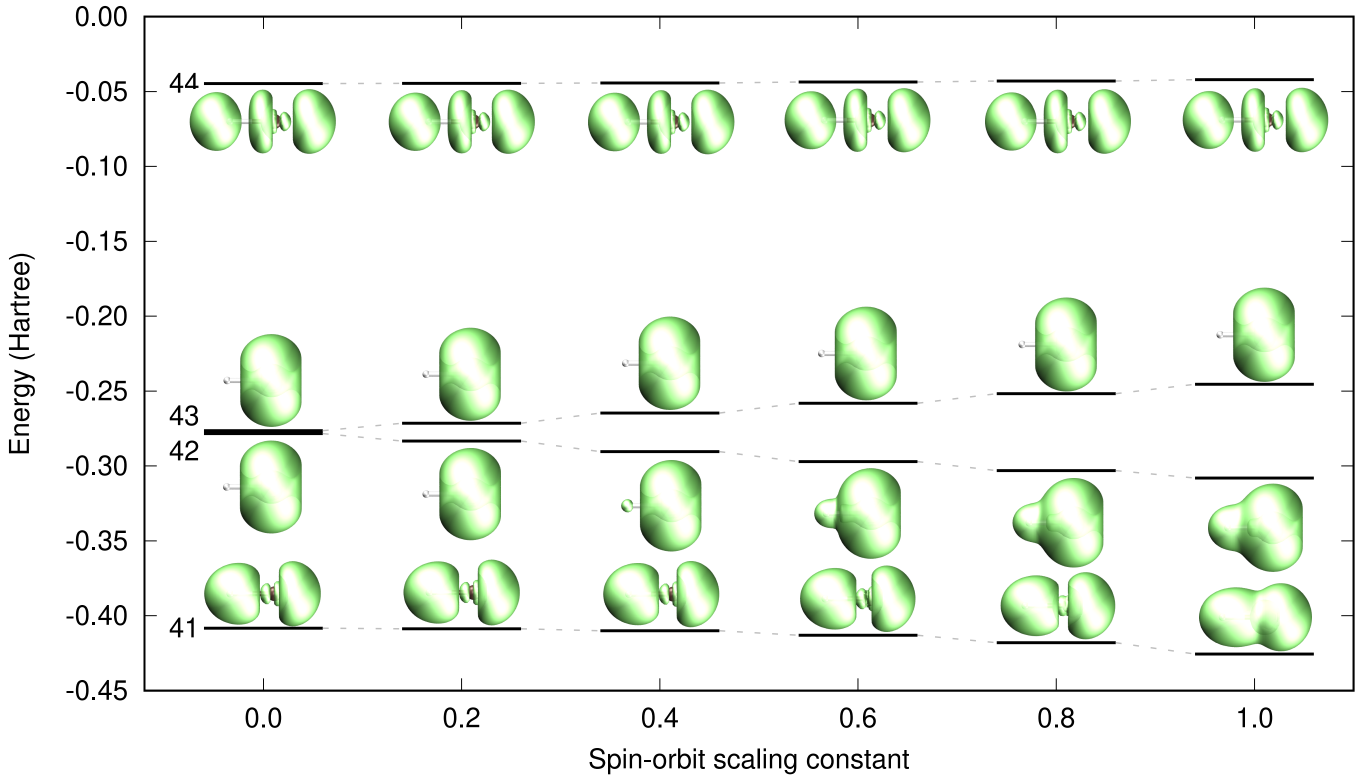

Figure S6: The frontier molecular orbitals of HAt upon increments of the spin-orbit scaling constant from 0.0 (SR-4c-DKS) to 1.0 (SOR-4c-DKS) in steps of 0.2.

Table S7: The Au–H and Au–X distances (in Å) of molecules AuH and **1a-1j** calculated at the 1c-ZORA-DFT/PBE0/QZ4P(Au),TZ2P(C,H,B,F,N,P,Si) and 2c-ZORA-DFT/PBE0/QZ4P(Au),TZ2P(C,H,B,F,N,P,Si) levels of theory, and the difference due to the inclusion of SOC

		1c-ZORA-DFT		2c-ZORA-DFT		Difference	
Molecule	X	d(Au–H)	d(Au–X)	d(Au–H)	d(Au–X)	d(Au–H)	d(Au–X)
AuH	–	1.534	–	–	–		
**1a**	F	1.562	2.045	1.561	2.037	−0.001	−0.008
**1b**	Cl	1.581	2.363	1.579	2.354	−0.002	−0.009
**1c**	Ph	1.633	2.078	1.628	2.068	−0.005	−0.010
**1d**	CH_3	1.636	2.118	1.631	2.110	−0.005	−0.008
**1e**	H	1.656	–	1.650	–	−0.006	–
**1f**	SiH_3	1.661	2.382	1.652	2.382	−0.009	0.000
**1g**	BH_2	1.701	2.069	1.693	2.060	−0.008	−0.009
**1h**	NH_3	1.559	2.179	1.559	2.167	0.000	−0.012
**1i**	CO	1.601	1.960	1.601	1.946	0.000	−0.014
**1j**	PH_3	1.605	2.305	1.603	2.290	−0.002	−0.015

Table S8: The Hg–H and Hg–X distances (in Å) of molecules **2a-2j** calculated at the 1c-ZORA-DFT/PBE0/QZ4P(Hg),TZ2P(C,H,B,F,N,P,Si) and 2c-ZORA-DFT/PBE0/QZ4P(Hg),TZ2P(C,H,B,F,N,P,Si) levels of theory, and the difference due to the inclusion of SOC

		1c-ZORA-DFT		2c-ZORA-DFT		Difference	
Molecule	X	d(Hg–H)	d(Hg–X)	d(Hg–H)	d(Hg–X)	d(Hg–H)	d(Hg–X)
**2a**	F	1.577	1.958	1.573	1.952	−0.004	−0.006
**2b**	Cl	1.600	2.290	1.595	2.282	−0.005	−0.008
**2c**	Ph	1.635	2.085	1.628	2.076	−0.007	−0.009
**2d**	CH_3	1.641	2.099	1.632	2.091	−0.009	−0.008
**2e**	H	1.646	–	1.637	–	−0.009	–
**2f**	SiH_3	1.665	2.464	1.656	2.453	−0.009	−0.011
**2g**	BH_2	1.669	2.164	1.660	2.152	−0.009	−0.012
**2h**	NH_3	1.585	2.169	1.581	2.157	−0.004	−0.012
**2i**	CO	1.589	2.205	1.586	2.183	−0.003	−0.022
**2j**	PH_3	1.608	2.472	1.604	2.454	−0.004	−0.018

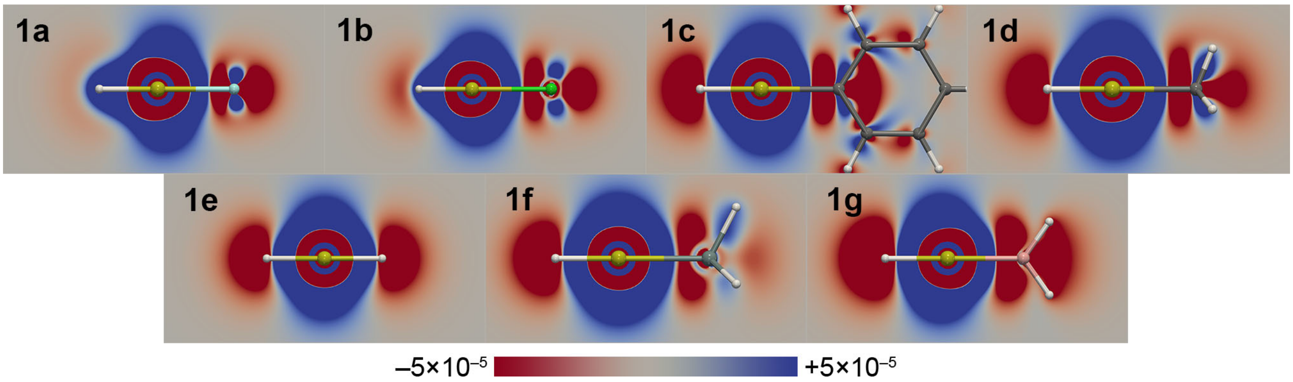

Figure S7: The SO-EDD in the molecular plane of molecules **1a-1g** calculated at the 4c-DKS level of theory. Red (blue) areas represent SO-induced depletion (concentration) of electron density. Color code: C, dark grey; H, white; Au, yellow; B, pink; Cl, green; F, light blue; Si, light grey.

Table S9: The M–H bond distance (in Å), the total NMR shielding constant (σ^{H} in ppm) and the net MICD strength circulating around the hydrogen atom (I^{H} in nA T^{-1}) calculated at the SR-4c-DKS and SOR-4c-DKS levels of theory for molecules **1h-1j** and **2h-2j**

Molecule	M–H distance	σ^{H}_{SR}	σ^{H}_{SOR}	$\Delta\sigma^{H}_{SO}$	I^{H}_{SR}	I^{H}_{SOR}	ΔI^{H}_{SO}
**1h**	1.559	+30.0	+30.0	+0.0	+5.50	+5.96	+0.46
**1i**	1.601	+30.9	+23.2	−7.7	+4.69	+3.91	−0.78
**1j**	1.605	+27.6	+27.1	−0.5	+4.56	+3.85	−0.71
**2h**	1.585	+26.3	+26.6	+0.3	+4.18	+4.18	+0.00
**2i**	1.589	+25.7	+25.4	−0.3	+4.07	+4.01	−0.06
**2j**	1.605	+25.5	+19.3	−6.2	+3.88	+3.29	−0.59

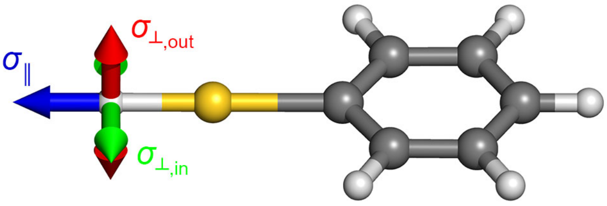

Figure S8: The orientation of the principal axes of the ^{1}H NMR shielding tensor for molecule **1c**.

Table S10: The total (σ_{iso}) NMR shielding constant of the hydride ligand of molecules AuH and **1a-1j** and its partition into perpendicular ($\sigma_{\perp,\text{out}}$, out of plane; $\sigma_{\perp,\text{in}}$, in plane) and parallel ($\sigma_{\parallel}$) components calculated at the SR-4c-DKS and SOR-4c-DKS levels of theory (all quantities are given in ppm)

	SR-4c-DKS				SOR-4c-DKS			
Molecule	σ_{iso}	$\sigma_{\perp,\text{out}}$	$\sigma_{\perp,\text{in}}$	$\sigma_{\parallel}$	σ_{iso}	$\sigma_{\perp,\text{out}}$	$\sigma_{\perp,\text{in}}$	$\sigma_{\parallel}$
AuH	31.000	17.227	17.227	58.546	51.840	49.481	49.481	56.557
**1a**	34.750	21.486	21.486	61.280	44.604	36.952	36.952	59.910
**1b**	32.302	18.224	18.224	60.458	36.339	24.914	24.914	59.191
**1c**	31.078	17.644	19.282	56.309	26.942	12.877	14.131	53.818
**1d**	31.023	17.734	17.734	57.601	25.944	11.071	11.071	55.690
**1e**	30.005	16.950	16.950	56.115	21.651	5.407	5.407	54.140
**1f**	28.612	15.193	15.193	55.451	16.008	−2.401	−2.401	52.827
**1g**	28.741	15.796	20.323	50.102	13.216	−11.698	9.581	41.766
**1h**	29.762	20.267	25.957	43.061	29.929	20.687	26.430	42.670
**1i**	30.905	17.725	17.725	57.266	23.153	7.220	7.220	55.017
**1j**	27.561	22.037	23.756	36.889	27.131	21.368	24.326	35.699

Table S11: The spin-orbit (SO) contribution to the total ($\sigma^{\text{SO}}_{\text{iso}}$) NMR shielding constant of the hydride ligand of molecules AuH and **1a-1j** and its partition into perpendicular ($\sigma^{\text{SO}}_{\perp,\text{out}}$, out of plane; $\sigma^{\text{SO}}_{\perp,\text{in}}$, in plane) and parallel ($\sigma^{\text{SO}}_{\parallel}$) components (all quantities are given in ppm)

Molecule	$\sigma^{\text{SO}}_{\text{iso}}$	$\sigma^{\text{SO}}_{\perp,\text{out}}$	$\sigma^{\text{SO}}_{\perp,\text{in}}$	$\sigma^{\text{SO}}_{\parallel}$
AuH	20.840	32.254	32.254	−1.989
**1a**	9.854	15.466	15.466	−1.370
**1b**	4.037	6.690	6.690	−1.267
**1c**	−4.136	−4.767	−5.151	−2.491
**1d**	−5.079	−6.663	−6.663	−1.911
**1e**	−8.354	−11.543	−11.543	−1.975
**1f**	−12.604	−17.594	−17.594	−2.624
**1g**	−15.525	−27.494	−10.742	−8.336
**1h**	0.167	0.420	0.473	−0.391
**1i**	−7.752	−10.505	−10.505	−2.249
**1j**	−0.430	−0.669	0.570	−1.190

Table S12: The diamagnetic component ($\sigma^{\text{d}}_{\text{iso}}$) of the NMR shielding constant of the hydride ligand of molecules AuH and **1a-1j** and its partition into perpendicular ($\sigma^{\text{d}}_{\perp,\text{out}}$, out of plane; $\sigma^{\text{d}}_{\perp,\text{in}}$, in plane) and parallel ($\sigma^{\text{d}}_{\parallel}$) components calculated at the SR-4c-DKS and SOR-4c-DKS levels of theory (all quantities are given in ppm)

	SR-4c-DKS				SOR-4c-DKS			
Molecule	$\sigma^{\text{d}}_{\text{iso}}$	$\sigma^{\text{d}}_{\perp,\text{out}}$	$\sigma^{\text{d}}_{\perp,\text{in}}$	$\sigma^{\text{d}}_{\parallel}$	$\sigma^{\text{d}}_{\text{iso}}$	$\sigma^{\text{d}}_{\perp,\text{out}}$	$\sigma^{\text{d}}_{\perp,\text{in}}$	$\sigma^{\text{d}}_{\parallel}$
AuH	28.057	12.811	12.811	58.550	28.268	13.105	13.105	58.594
**1a**	32.060	17.422	17.422	61.334	32.246	17.685	17.685	61.367
**1b**	31.731	17.348	17.348	60.497	31.850	17.511	17.511	60.527
**1c**	34.294	24.491	19.475	58.916	34.357	24.656	19.500	58.914
**1d**	31.734	18.732	18.732	57.738	31.724	18.716	18.716	57.740
**1e**	31.060	18.519	18.519	56.141	31.043	18.492	18.492	56.146
**1f**	30.495	17.640	17.640	56.204	30.418	17.527	17.527	56.200
**1g**	30.200	18.295	17.873	54.432	30.048	18.051	17.689	54.404
**1h**	28.279	19.070	21.494	44.272	28.277	19.269	21.301	44.261
**1i**	30.227	16.686	16.686	57.308	30.184	16.634	16.634	57.285
**1j**	27.917	20.321	22.186	41.245	27.933	20.531	22.189	41.078

Table S13: The spin-orbit (SO) contribution to the diamagnetic ($\sigma_{\mathrm{iso}}^{\mathrm{d,SO}}$) NMR shielding constant of the hydride ligand of molecules AuH and **1a-1j** and its partition into perpendicular ($\sigma_{\perp,\mathrm{out}}^{\mathrm{d,SO}}$, out of plane; $\sigma_{\perp,\mathrm{in}}^{\mathrm{d,SO}}$, in plane) and parallel ($\sigma_{\parallel}^{\mathrm{d,SO}}$) components (all quantities are given in ppm)

Molecule	$\sigma_{\mathrm{iso}}^{\mathrm{d,SO}}$	$\sigma_{\perp,\mathrm{out}}^{\mathrm{d,SO}}$	$\sigma_{\perp,\mathrm{in}}^{\mathrm{d,SO}}$	$\sigma_{\parallel}^{\mathrm{d,SO}}$
AuH	0.211	0.294	0.294	0.044
**1a**	0.186	0.263	0.263	0.033
**1b**	0.119	0.163	0.163	0.030
**1c**	0.063	0.165	0.025	−0.002
**1d**	−0.010	−0.016	−0.016	0.002
**1e**	−0.017	−0.027	−0.027	0.005
**1f**	−0.077	−0.113	−0.113	−0.004
**1g**	−0.152	−0.244	−0.184	−0.028
**1h**	−0.002	0.199	−0.193	−0.011
**1i**	−0.043	−0.052	−0.052	−0.023
**1j**	0.016	0.210	0.003	−0.167

Table S14: The paramagnetic component ($\sigma_{\mathrm{iso}}^{\mathrm{p}}$) of the NMR shielding constant of the hydride ligand of molecules AuH and **1a-1j** and its partition into perpendicular ($\sigma_{\perp,\mathrm{out}}^{\mathrm{p}}$, out of plane; $\sigma_{\perp,\mathrm{in}}^{\mathrm{p}}$, in plane) and parallel ($\sigma_{\parallel}^{\mathrm{p}}$) components calculated at the SR-4c-DKS and SOR-4c-DKS levels of theory (all quantities are given in ppm)

	SR-4c-DKS				SOR-4c-DKS			
Molecule	$\sigma_{\mathrm{iso}}^{\mathrm{p}}$	$\sigma_{\perp,\mathrm{out}}^{\mathrm{p}}$	$\sigma_{\perp,\mathrm{in}}^{\mathrm{p}}$	$\sigma_{\parallel}^{\mathrm{p}}$	$\sigma_{\mathrm{iso}}^{\mathrm{p}}$	$\sigma_{\perp,\mathrm{out}}^{\mathrm{p}}$	$\sigma_{\perp,\mathrm{in}}^{\mathrm{p}}$	$\sigma_{\parallel}^{\mathrm{p}}$
AuH	2.942	4.416	4.416	−0.005	23.571	36.376	36.376	−0.038
**1a**	2.691	4.063	4.063	−0.054	12.358	19.266	19.266	−1.458
**1b**	0.571	0.876	0.876	−0.039	4.490	7.403	7.403	−1.336
**1c**	−3.215	−6.847	−0.193	−2.606	−7.415	−11.779	−5.369	−5.096
**1d**	−0.711	−0.997	−0.997	−0.137	−5.780	−7.644	−7.644	−2.050
**1e**	−1.055	−1.569	−1.569	−0.025	−9.392	−13.085	−13.085	−2.006
**1f**	−1.883	−2.447	−2.447	−0.753	−14.410	−19.928	−19.928	−3.373
**1g**	−1.459	−2.499	2.451	−4.330	−16.832	−29.748	−8.108	−12.638
**1h**	1.483	1.196	4.463	−1.211	1.652	1.418	5.129	−1.591
**1i**	0.678	1.038	1.038	−0.042	−7.032	−9.413	−9.413	−2.268
**1j**	−0.357	1.717	1.569	−4.356	−0.802	0.837	2.136	−5.379

Table S15: The spin-orbit (SO) contribution to the paramagnetic ($\sigma_{\mathrm{iso}}^{\mathrm{p,SO}}$) NMR shielding constant of the hydride ligand of molecules AuH and **1a-1j** and its partition into perpendicular ($\sigma_{\perp,\mathrm{out}}^{\mathrm{p,SO}}$, out of plane; $\sigma_{\perp,\mathrm{in}}^{\mathrm{p,SO}}$, in plane) and parallel ($\sigma_{\parallel}^{\mathrm{p,SO}}$) components (all quantities are given in ppm)

Molecule	$\sigma_{\mathrm{iso}}^{\mathrm{p,SO}}$	$\sigma_{\perp,\mathrm{out}}^{\mathrm{p,SO}}$	$\sigma_{\perp,\mathrm{in}}^{\mathrm{p,SO}}$	$\sigma_{\parallel}^{\mathrm{p,SO}}$
AuH	20.629	31.960	31.960	−2.033
**1a**	9.667	15.203	15.203	−1.404
**1b**	3.919	6.527	6.527	−1.297
**1c**	−4.200	−4.932	−5.176	−2.490
**1d**	−5.069	−6.647	−6.647	−1.913
**1e**	−8.337	−11.516	−11.516	−1.981
**1f**	−12.527	−17.481	−17.481	−2.620
**1g**	−15.373	−27.249	−10.559	−8.308
**1h**	0.169	0.222	0.666	−0.380
**1i**	−7.710	−10.451	−10.451	−2.226
**1j**	−0.445	−0.880	6.492	−1.023

Table S16: The total (σ_{iso}) NMR shielding constant of the hydride ligand of molecules **2a-2j** and its partition into perpendicular ($\sigma_{\perp,\text{out}}$, out of plane; $\sigma_{\perp,\text{in}}$, in plane) and parallel ($\sigma_{\parallel}$) components calculated at the SR-4c-DKS and SOR-4c-DKS levels of theory (all quantities are given in ppm)

	SR-4c-DKS				SOR-4c-DKS			
Molecule	σ_{iso}	$\sigma_{\perp,\text{out}}$	$\sigma_{\perp,\text{in}}$	$\sigma_{\parallel}$	σ_{iso}	$\sigma_{\perp,\text{out}}$	$\sigma_{\perp,\text{in}}$	$\sigma_{\parallel}$
**2a**	28.527	14.595	14.595	56.391	30.157	17.661	17.661	55.151
**2b**	27.268	13.008	13.008	55.787	23.186	7.592	7.592	54.373
**2c**	27.417	13.636	14.619	53.997	14.732	−3.529	−3.016	50.741
**2d**	27.318	13.785	13.785	54.382	11.159	−8.933	−8.933	51.342
**2e**	27.486	14.506	14.506	53.447	10.776	−8.954	−8.953	50.235
**2f**	26.234	12.929	12.930	52.844	2.372	−20.641	−20.637	48.395
**2g**	26.967	13.462	16.085	51.355	3.287	−26.436	−6.798	43.094
**2h**	26.279	12.278	12.298	54.261	26.576	13.376	13.377	52.974
**2i**	25.687	11.879	11.879	53.302	25.412	12.090	12.090	52.055
**2j**	25.540	11.670	11.670	53.280	19.302	3.221	3.222	51.463

Table S17: The spin-orbit (SO) contribution to the total ($\sigma^{\text{SO}}_{\text{iso}}$) NMR shielding constant of the hydride ligand of molecules **2a-2j** and its partition into perpendicular ($\sigma^{\text{SO}}_{\perp,\text{out}}$, out of plane; $\sigma^{\text{SO}}_{\perp,\text{in}}$, in plane) and parallel ($\sigma^{\text{SO}}_{\parallel}$) components (all quantities are given in ppm)

Molecule	$\sigma^{\text{SO}}_{\text{iso}}$	$\sigma^{\text{SO}}_{\perp,\text{out}}$	$\sigma^{\text{SO}}_{\perp,\text{in}}$	$\sigma^{\text{SO}}_{\parallel}$
**2a**	1.630	3.066	3.066	−1.240
**2b**	−4.082	−5.416	−5.416	−1.414
**2c**	−12.685	−17.165	−17.635	−3.256
**2d**	−16.159	−22.718	−22.718	−3.040
**2e**	−16.710	−23.460	−23.459	−3.212
**2f**	−23.862	−33.570	−33.567	−4.449
**2g**	−23.680	−39.898	−22.883	−8.261
**2h**	0.297	1.098	1.079	−1.287
**2i**	−0.275	0.211	0.211	−1.247
**2j**	−6.238	−8.449	−8.448	−1.817

Table S18: The diamagnetic component ($\sigma^{\text{d}}_{\text{iso}}$) of the NMR shielding constant of the hydride ligand of molecules **2a-2j** and its partition into perpendicular ($\sigma^{\text{d}}_{\perp,\text{out}}$, out of plane; $\sigma^{\text{d}}_{\perp,\text{in}}$, in plane) and parallel ($\sigma^{\text{d}}_{\parallel}$) components calculated at the SR-4c-DKS and SOR-4c-DKS levels of theory (all quantities are given in ppm)

	SR-4c-DKS				SOR-4c-DKS			
Molecule	$\sigma^{\text{d}}_{\text{iso}}$	$\sigma^{\text{d}}_{\perp,\text{out}}$	$\sigma^{\text{d}}_{\perp,\text{in}}$	$\sigma^{\text{d}}_{\parallel}$	$\sigma^{\text{d}}_{\text{iso}}$	$\sigma^{\text{d}}_{\perp,\text{out}}$	$\sigma^{\text{d}}_{\perp,\text{in}}$	$\sigma^{\text{d}}_{\parallel}$
**2a**	27.936	13.698	13.698	56.411	27.965	13.735	13.735	56.424
**2b**	27.968	14.051	14.051	55.801	27.946	14.015	14.015	55.807
**2c**	31.024	21.906	15.564	55.601	30.904	21.740	15.402	55.572
**2d**	29.060	16.364	16.364	54.451	28.920	16.171	16.171	54.419
**2e**	28.334	15.774	15.774	53.453	28.179	15.561	15.561	53.414
**2f**	28.387	15.923	15.924	53.313	28.196	15.659	15.659	53.271
**2g**	28.416	16.409	15.749	53.089	28.192	16.098	15.453	53.026
**2h**	26.652	12.842	12.841	54.274	26.656	12.841	12.839	54.287
**2i**	25.044	10.909	10.909	53.314	25.041	10.898	10.898	53.326
**2j**	26.334	12.675	12.675	53.652	26.281	12.593	12.593	53.655

Table S19: The spin-orbit (SO) contribution to the diamagnetic ($\sigma_{\text{iso}}^{\text{d,SO}}$) NMR shielding constant of the hydride ligand of molecules **2a-2j** and its partition into perpendicular ($\sigma_{\perp,\text{out}}^{\text{d,SO}}$, out of plane; $\sigma_{\perp,\text{in}}^{\text{d,SO}}$, in plane) and parallel ($\sigma_{\parallel}^{\text{d,SO}}$) components (all quantities are given in ppm)

Molecule	$\sigma_{\text{iso}}^{\text{d,SO}}$	$\sigma_{\perp,\text{out}}^{\text{d,SO}}$	$\sigma_{\perp,\text{in}}^{\text{d,SO}}$	$\sigma_{\parallel}^{\text{d,SO}}$
**2a**	0.029	0.037	0.037	0.013
**2b**	−0.022	−0.036	−0.036	0.006
**2c**	−0.120	−0.166	−0.162	−0.029
**2d**	−0.140	−0.193	−0.193	−0.032
**2e**	−0.155	−0.213	−0.213	−0.039
**2f**	−0.191	−0.264	−0.265	−0.042
**2g**	−0.224	−0.311	−0.296	−0.063
**2h**	0.004	−0.001	−0.002	0.013
**2i**	−0.003	−0.011	−0.011	0.012
**2j**	−0.053	−0.082	−0.082	0.003

Table S20: The paramagnetic component ($\sigma_{\text{iso}}^{\text{p}}$) of the NMR shielding constant of the hydride ligand of molecules **2a-2j** and its partition into perpendicular ($\sigma_{\perp,\text{out}}^{\text{p}}$, out of plane; $\sigma_{\perp,\text{in}}^{\text{p}}$, in plane) and parallel ($\sigma_{\parallel}^{\text{p}}$) components calculated at the SR-4c-DKS and SOR-4c-DKS levels of theory (all quantities are given in ppm)

	SR-4c-DKS				SOR-4c-DKS			
Molecule	$\sigma_{\text{iso}}^{\text{p}}$	$\sigma_{\perp,\text{out}}^{\text{p}}$	$\sigma_{\perp,\text{in}}^{\text{p}}$	$\sigma_{\parallel}^{\text{p}}$	$\sigma_{\text{iso}}^{\text{p}}$	$\sigma_{\perp,\text{out}}^{\text{p}}$	$\sigma_{\perp,\text{in}}^{\text{p}}$	$\sigma_{\parallel}^{\text{p}}$
**2a**	0.591	0.897	0.897	−0.020	2.193	3.926	3.926	−1.273
**2b**	−0.700	−1.043	−1.043	−0.014	−4.760	−6.423	−6.423	−1.434
**2c**	−3.606	−8.270	−0.945	−1.604	−16.172	−25.269	−18.418	−4.831
**2d**	−1.742	−2.579	−2.579	−0.069	−17.761	−25.103	−25.103	−3.077
**2e**	−0.848	−1.269	−1.269	−0.005	−17.403	−24.515	−24.514	−3.179
**2f**	−2.152	−2.994	−2.994	−0.469	−25.824	−36.300	−6.296	−4.876
**2g**	−1.448	−2.946	0.336	−1.734	−24.906	−42.534	−22.251	−9.931
**2h**	−0.373	−0.564	−0.543	−0.013	−0.080	0.536	0.538	−1.313
**2i**	0.643	0.971	0.971	−0.012	0.371	1.192	1.192	−1.270
**2j**	−0.794	−1.005	−1.005	−0.372	−6.979	−9.372	−9.371	−2.193

Table S21: The spin-orbit (SO) contribution to the paramagnetic ($\sigma_{\text{iso}}^{\text{p,SO}}$) NMR shielding constant of the hydride ligand of molecules **2a-2j** and its partition into perpendicular ($\sigma_{\perp,\text{out}}^{\text{p,SO}}$, out of plane; $\sigma_{\perp,\text{in}}^{\text{p,SO}}$, in plane) and parallel ($\sigma_{\parallel}^{\text{p,SO}}$) components (all quantities are given in ppm)

Molecule	$\sigma_{\text{iso}}^{\text{p,SO}}$	$\sigma_{\perp,\text{out}}^{\text{p,SO}}$	$\sigma_{\perp,\text{in}}^{\text{p,SO}}$	$\sigma_{\parallel}^{\text{p,SO}}$
**2a**	1.602	3.029	3.029	−1.253
**2b**	−4.060	−5.380	−5.380	−1.420
**2c**	−12.566	−16.999	−17.473	−3.227
**2d**	−16.019	−22.524	−22.524	−3.008
**2e**	−16.555	−23.246	−23.245	−3.174
**2f**	−23.672	−33.306	−33.302	−4.407
**2g**	−23.458	−39.588	−22.587	−8.197
**2h**	0.293	1.100	1.081	−1.300
**2i**	−0.272	0.221	0.221	−1.258
**2j**	−6.185	−8.367	−8.366	−1.821

Table S22: The net MICD strength circulating around the hydrogen atom and its partition into diatropic and paratropic components calculated at the SR-4c-DKS and SOR-4c-DKS levels of theory of molecules AuH and **1a-1j** when the external magnetic field vector is perpendicular to the molecular plane (all quantities are given in nA T^{-1})

	SR-4c-DKS			SOR-4c-DKS		
Molecule	Net	Diatropic	Paratropic	Net	Diatropic	Paratropic
AuH	+5.96	+5.96	−0.00	+7.76	+7.76	−0.00
**1a**	+6.41	+6.41	−0.00	+7.17	+7.17	−0.00
**1b**	+5.65	+5.65	−0.00	+5.97	+5.97	−0.00
**1c**	+5.23	+5.23	−0.00	+4.83	+4.83	−0.00
**1d**	+5.06	+5.06	−0.00	+4.57	+4.57	−0.00
**1e**	+4.85	+4.85	−0.00	+4.08	+4.08	−0.00
**1f**	+4.47	+4.47	−0.00	+3.32	+3.33	−0.01
**1g**	+4.51	+4.51	−0.00	+2.72	+2.83	−0.11
**1h**	+5.50	+5.50	−0.00	+5.96	+5.96	−0.00
**1i**	+4.69	+4.69	−0.00	+3.91	+3.91	−0.00
**1j**	+4.56	+4.56	−0.00	+3.85	+3.85	−0.00

Table S23: The spin-orbit contribution to the net MICD strength circulating around the hydrogen atom and its partition into diatropic and paratropic components of molecules AuH and **1a-1j** when the external magnetic field vector is perpendicular to the molecular plane (all quantities are given in nA T^{-1})

Molecule	Net	Diatropic	Paratropic
AuH	+1.80	+1.80	−0.00
**1a**	+0.76	+0.76	−0.00
**1b**	+0.32	+0.32	−0.00
**1c**	−0.40	−0.40	−0.00
**1d**	−0.49	−0.49	−0.00
**1e**	−0.77	−0.77	−0.00
**1f**	−1.15	−1.14	−0.01
**1g**	−1.79	−1.68	−0.11
**1h**	+0.46	+0.46	−0.00
**1i**	−0.78	−0.78	−0.00
**1j**	−0.71	−0.71	−0.00

Table S24: The net MICD strength circulating around the hydrogen atom and its partition into diatropic and paratropic components calculated at the SR-4c-DKS and SOR-4c-DKS levels of theory of molecules **2a-2j** when the external magnetic field vector is perpendicular to the molecular plane (all quantities are given in nA T^{-1})

	SR-4c-DKS			SOR-4c-DKS		
Molecule	Net	Diatropic	Paratropic	Net	Diatropic	Paratropic
**2a**	+4.63	+4.63	−0.00	+4.75	+4.75	−0.00
**2b**	+4.23	+4.23	−0.00	+3.85	+3.85	−0.00
**2c**	+4.14	+4.14	−0.00	+3.03	+3.03	−0.00
**2d**	+3.92	+3.92	−0.00	+2.48	+2.50	−0.02
**2e**	+3.99	+3.99	−0.00	+2.48	+2.51	−0.03
**2f**	+3.74	+3.74	−0.00	+1.65	+1.84	−0.19
**2g**	+3.64	+3.64	−0.00	+1.11	+1.59	−0.49
**2h**	+4.18	+4.18	−0.00	+4.18	+4.18	−0.00
**2i**	+4.07	+4.07	−0.00	+4.01	+4.01	−0.00
**2j**	+3.88	+3.88	−0.00	+3.29	+3.29	−0.00

Table S25: The spin-orbit contribution to the net MICD strength circulating around the hydrogen atom and its partition into diatropic and paratropic components of molecules **2a-2j** when the external magnetic field vector is perpendicular to the molecular plane (all quantities are given in nA T^{-1})

Molecule	Net	Diatropic	Paratropic
**2a**	+0.12	+0.12	−0.00
**2b**	−0.38	−0.38	−0.00
**2c**	−1.11	−1.11	−0.00
**2d**	−1.44	−1.42	−0.02
**2e**	−1.51	−1.48	−0.03
**2f**	−2.09	−1.90	−0.19
**2g**	−2.53	−2.05	−0.49
**2h**	+0.00	+0.00	−0.00
**2i**	−0.06	−0.06	−0.00
**2j**	−0.59	−0.59	−0.00

Table S26: The total ligand ^{1}H NMR shielding constant (σ^{H} in ppm), and the net MICD strength circulating around the hydrogen atom (I^{H} in nA T^{-1}) calculated at the SR-4c Dirac–Hartree–Fock and SOR-4c Dirac–Hartree–Fock levels of theory for molecules AuH, and **1a-1g** (the values calculated at the SR-4c-DKS and SOR-4c-DKS levels of theory are included for purposes of comparison)

4c Dirac–Hartree–Fock level of theory						
Molecule	$\sigma^{\mathrm{H}}_{\mathrm{SR}}$	$\sigma^{\mathrm{H}}_{\mathrm{SOR}}$	$\Delta\sigma^{\mathrm{H}}_{\mathrm{SO}}$	$I^{\mathrm{H}}_{\mathrm{SR}}$	$I^{\mathrm{H}}_{\mathrm{SOR}}$	$\Delta I^{\mathrm{H}}_{\mathrm{SO}}$
AuH	+30.459	+51.976	+21.517	+5.93	+7.75	+1.82
**1a**	+34.384	+47.440	+13.056	+6.43	+7.51	+1.08
**1b**	+32.386	+37.786	+5.400	+5.90	+6.33	+0.43
**1c**	+31.318	+28.929	−2.389	+5.49	+5.23	−0.26
**1d**	+31.254	+27.598	−3.656	+5.38	+5.02	−0.36
**1e**	+30.296	+23.421	−6.875	+5.18	+4.55	−0.63
**1f**	+29.232	+16.624	−12.608	+4.83	+3.71	−1.12
**1g**	+29.546	+18.689	−10.857	+4.72	+3.07	−1.65
4c-DKS/PBE0 level of theory						
Molecule	$\sigma^{\mathrm{H}}_{\mathrm{SR}}$	$\sigma^{\mathrm{H}}_{\mathrm{SOR}}$	$\Delta\sigma^{\mathrm{H}}_{\mathrm{SO}}$	$I^{\mathrm{H}}_{\mathrm{SR}}$	$I^{\mathrm{H}}_{\mathrm{SOR}}$	$\Delta I^{\mathrm{H}}_{\mathrm{SO}}$
AuH	+31.055	+51.218	+20.163	+5.96	+7.76	+1.80
**1a**	+34.750	+44.604	+9.854	+6.41	+7.17	+0.76
**1b**	+32.302	+36.339	+4.037	+5.65	+5.97	+0.32
**1c**	+31.083	+26.949	−4.134	+5.23	+4.83	−0.40
**1d**	+30.005	+21.651	−8.354	+5.06	+4.57	−0.49
**1e**	+31.023	+25.944	−5.079	+4.85	+4.08	−0.77
**1f**	+28.651	+16.308	−12.343	+4.47	+3.33	−1.14
**1g**	+28.739	+13.209	−15.530	+4.51	+2.72	−1.79

Table S27: The total ligand ^{1}H NMR shielding constant (σ^{H} in ppm), and the net MICD strength circulating around the hydrogen atom (I^{H} in nA T^{-1}) calculated at the non-relativistic (NR) 4c-DKS level of theory for molecules AuH, and **1a-1g** (the values calculated at the SR-4c-DKS and SOR-4c-DKS levels of theory are included for purposes of comparison)

^{1}H NMR shielding constants					
Molecule	$\sigma^{\mathrm{H}}_{\mathrm{NR}}$	$\sigma^{\mathrm{H}}_{\mathrm{SR}}$	$\sigma^{\mathrm{H}}_{\mathrm{SOR}}$	$\Delta\sigma^{\mathrm{H}}_{\mathrm{SR}}$ ^{a}	$\Delta\sigma^{\mathrm{H}}_{\mathrm{SO}}$ ^{b}
AuH	+30.977	+31.055	+51.218	+0.078	+20.163
**1a**	+34.806	+34.750	+44.604	−0.056	+9.854
**1b**	+32.829	+32.302	+36.339	−0.527	+4.037
**1c**	+31.401	+31.083	+26.949	−0.318	−4.134
**1d**	+30.230	+30.005	+21.651	−0.225	−8.354
**1e**	+31.398	+31.023	+25.944	−0.375	−5.079
**1f**	+29.094	+28.651	+16.308	−0.443	−12.343
**1g**	+29.334	+28.739	+13.209	−0.595	−15.530
MICD strengths					
Molecule	$I^{\mathrm{H}}_{\mathrm{NR}}$	$I^{\mathrm{H}}_{\mathrm{SR}}$	$I^{\mathrm{H}}_{\mathrm{SOR}}$	$\Delta I^{\mathrm{H}}_{\mathrm{SR}}$ ^{a}	$\Delta I^{\mathrm{H}}_{\mathrm{SO}}$ ^{b}
AuH	+5.68	+5.96	+7.76	+0.28	+1.80
**1a**	+6.36	+6.41	+7.17	+0.05	+0.76
**1b**	+5.84	+5.65	+5.97	−0.19	+0.32
**1c**	+5.36	+5.23	+4.83	−0.13	−0.40
**1d**	+5.00	+5.06	+4.57	+0.06	−0.49
**1e**	+5.25	+4.85	+4.08	−0.40	−0.77
**1f**	+4.68	+4.47	+3.33	−0.21	−1.14
**1g**	+4.52	+4.51	+2.72	−0.01	−1.79

^{a} $\Delta\sigma^{\mathrm{H}}_{\mathrm{SR}} = \sigma^{\mathrm{H}}_{\mathrm{SR}} - \sigma^{\mathrm{H}}_{\mathrm{NR}}$; $\Delta I^{\mathrm{H}}_{\mathrm{SR}} = I^{\mathrm{H}}_{\mathrm{SR}} - I^{\mathrm{H}}_{\mathrm{NR}}$
^{b} $\Delta\sigma^{\mathrm{H}}_{\mathrm{SO}} = \sigma^{\mathrm{H}}_{\mathrm{SOR}} - \sigma^{\mathrm{H}}_{\mathrm{SR}}$; $\Delta I^{\mathrm{H}}_{\mathrm{SO}} = I^{\mathrm{H}}_{\mathrm{SOR}} - I^{\mathrm{H}}_{\mathrm{SR}}$

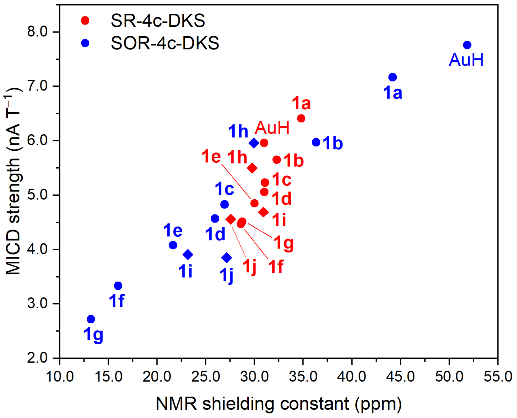

Figure S9: The MICD strength as a function of the total NMR shielding constant of AuH and molecules **1a-1j**, calculated at the SR-4c-DKS (red) and SOR-4c-DKS (blue) levels of theory. Molecules **1h-1j** with a different charge state are shown with diamonds.

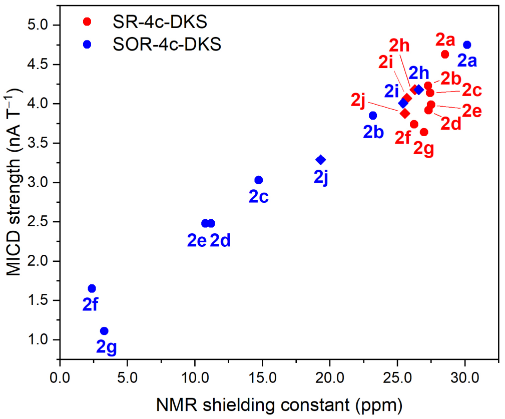

Figure S10: The MICD strength as a function of the total NMR shielding constant of molecules **2a-2j**, calculated at the SR-4c-DKS (red) and SOR-4c-DKS (blue) levels of theory. Molecules **2h-2j** with a different charge state are shown with diamonds.

Table S28: The contribution of the electronic TLI to the MICD strength of molecules **1a-1j** calculated at the SR-4c-DKS [$I^{\mathrm{H}}_{\mathrm{SR}}$(electronic)] and SOR-4c-DKS levels of theory [$I^{\mathrm{H}}_{\mathrm{SOR}}$(electronic), all quantities are given in nA T^{-1}]

Molecule	Au–H bond length (Å)	$I^{\mathrm{H}}_{\mathrm{SR}}$(tot) ^{a}	$I^{\mathrm{H}}_{\mathrm{SR}}$(str) ^{b}	$I^{\mathrm{H}}_{\mathrm{SR}}$(ele) ^{c}	$I^{\mathrm{H}}_{\mathrm{SOR}}$(tot) ^{a}	$I^{\mathrm{H}}_{\mathrm{SOR}}$(str) ^{b}	$I^{\mathrm{H}}_{\mathrm{SOR}}$(ele) ^{c}
**1a**	1.562	+6.41	+5.79	+0.62	+7.17	+7.61	−0.44
**1b**	1.581	+5.65	+5.68	−0.03	+5.97	+7.52	−1.55
**1c**	1.633	+5.23	+5.41	−0.18	+4.83	+7.27	−2.44
**1d**	1.636	+5.06	+5.39	−0.33	+4.57	+7.26	−2.69
**1e**	1.656	+4.85	+5.30	−0.45	+4.08	+7.17	−3.09
**1f**	1.661	+4.47	+5.27	−0.80	+3.32	+7.15	−3.83
**1g**	1.701	+4.51	+5.09	−0.58	+2.72	+6.98	−4.26
**1h**	1.559	+5.50	+5.81	−0.31	+5.96	+7.63	−1.67
**1i**	1.601	+4.69	+5.57	−0.88	+3.91	+7.42	−3.51
**1j**	1.605	+4.56	+5.55	−0.99	+3.85	+7.40	−3.55

^{a} I^{H}(total) is obtained by plane integration of molecules **1a-1g**

^{b} I^{H}(structural) is obtained by plane integration of AuH at the Au−H equilibrium bond length of molecules **1a-1g**

^{c} I^{H}(electronic) = I^{H}(total) − I^{H}(structural)

Table S29: The spin-orbit contribution of the electronic TLI to the MICD strength of molecules **1a-1j** [$\Delta I^{\mathrm{H}}_{\mathrm{SO}}$(electronic), all quantities are given in nA T^{-1}]

Molecule	Au–H bond length (Å)	$\Delta I^{\mathrm{H}}_{\mathrm{SO}}$(total) ^{a}	$\Delta I^{\mathrm{H}}_{\mathrm{SO}}$(structural) ^{b}	$\Delta I^{\mathrm{H}}_{\mathrm{SO}}$(electronic) ^{c}
**1a**	1.562	+0.76	+1.82	−1.06
**1b**	1.581	+0.32	+1.84	−1.52
**1c**	1.633	−0.40	+1.86	−2.26
**1d**	1.636	−0.49	+1.87	−2.36
**1e**	1.656	−0.77	+1.87	−2.64
**1f**	1.661	−1.15	+1.88	−3.03
**1g**	1.701	−1.79	+1.89	−3.68
**1h**	1.559	+0.46	+1.82	−1.36
**1i**	1.601	−0.78	+1.85	−2.63
**1j**	1.605	−0.71	+1.85	−2.56

^{a} $\Delta I^{\mathrm{H}}_{\mathrm{SO}}$(total) = $I^{\mathrm{H}}_{\mathrm{SOR}}$(total) − $I^{\mathrm{H}}_{\mathrm{SR}}$(total)

^{b} $\Delta I^{\mathrm{H}}_{\mathrm{SO}}$(structural) = $I^{\mathrm{H}}_{\mathrm{SOR}}$(structural) − $I^{\mathrm{H}}_{\mathrm{SR}}$(structural)

^{c} $\Delta I^{\mathrm{H}}_{\mathrm{SO}}$(electronic) = $\Delta I^{\mathrm{H}}_{\mathrm{SO}}$(total) − $\Delta I^{\mathrm{H}}_{\mathrm{SO}}$(structural)

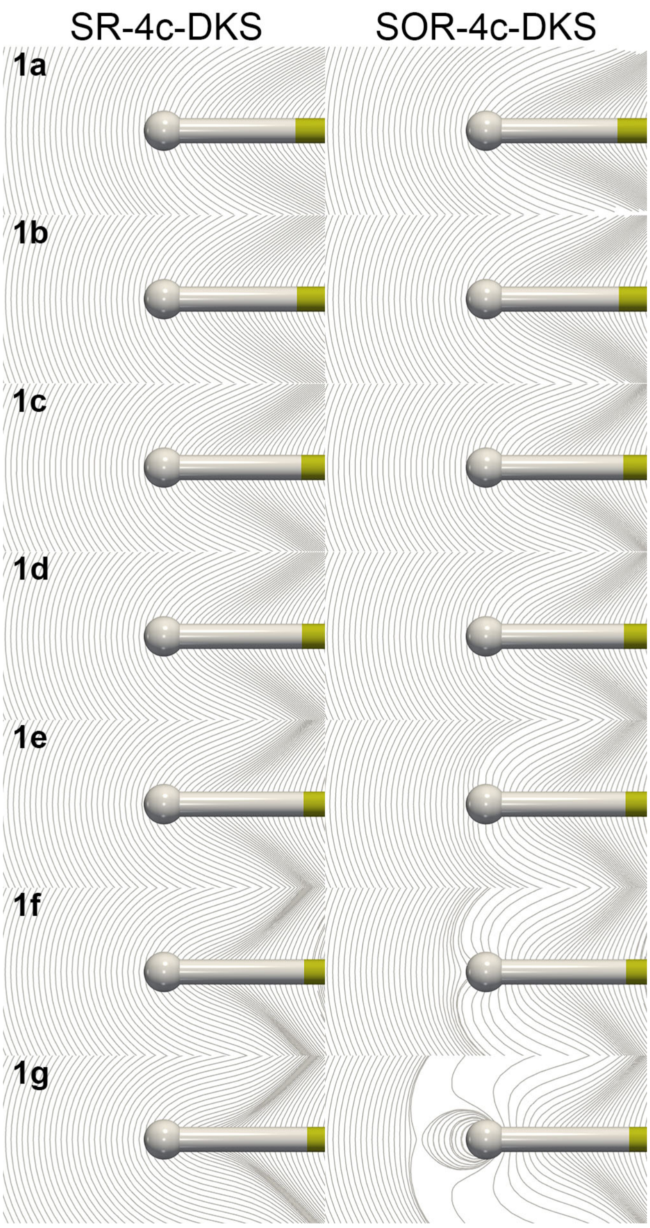

Figure S11: A detail of the MICD delocalization pathways circulating around H of molecules **1a** to **1g** at the SR-4c-DKS (left) and SOR-4c-DKS (right) levels of theory.

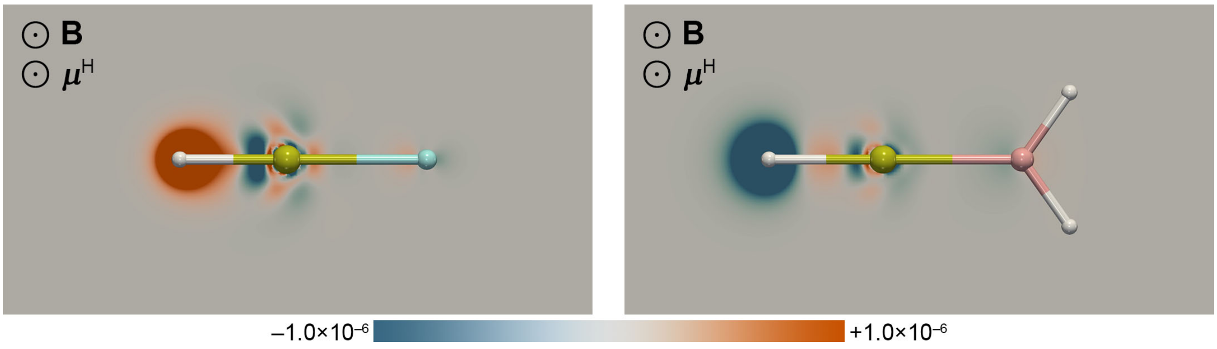

Figure S12: The positive (in orange) and negative (in blue) spin-orbit contributions to the magnetic shielding density of H of molecules **1a** (left) and **1g** (right). Color code: H, white; Au, yellow; B, pink; F, cyan.

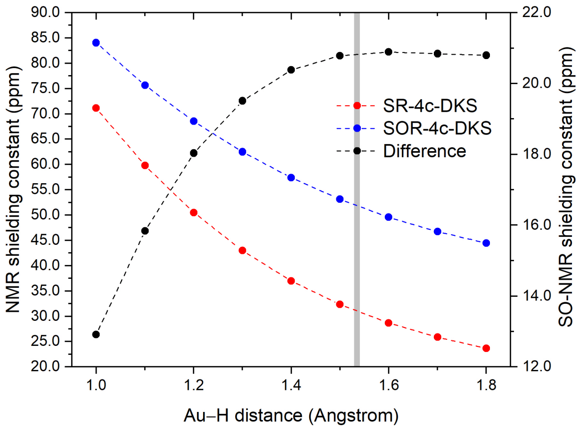

Figure S13: The total NMR shielding constant at the SR-4c-DKS and SOR-4c-DKS levels of theory and the derived SO-HALA NMR shielding constant as a function of the Au–H distance for AuH. The gray bar indicates the optimized equilibrium distance of AuH.

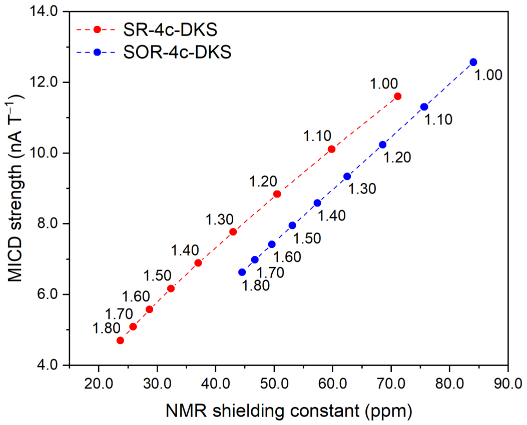

Figure S14: The MICD strength as a function of the total NMR shielding constant calculated at the SR-4c-DKS and SOR-4c-DKS levels of theory for AuH. The corresponding Au–H distances are indicated in the graph.

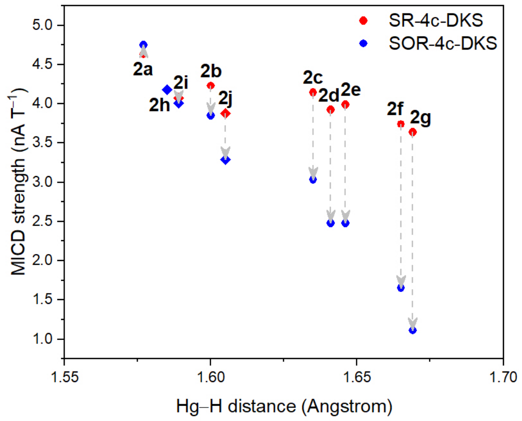

Figure S15: The MICD strength as a function of the Hg–H distance for molecules **2a-2j**, calculated at the SR-4c-DKS (red points) and SOR-4c-DKS (blue points) levels of theory. The gray dashed arrows indicate the SO-induced departure of the MICD strengths.

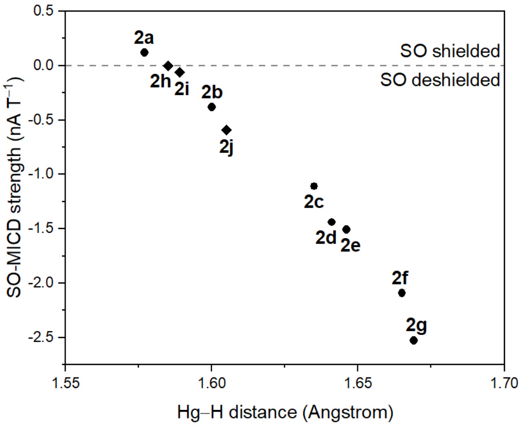

Figure S16: The SO-MICD strength as a function of the Hg–H distance for molecules **2a-2j**.

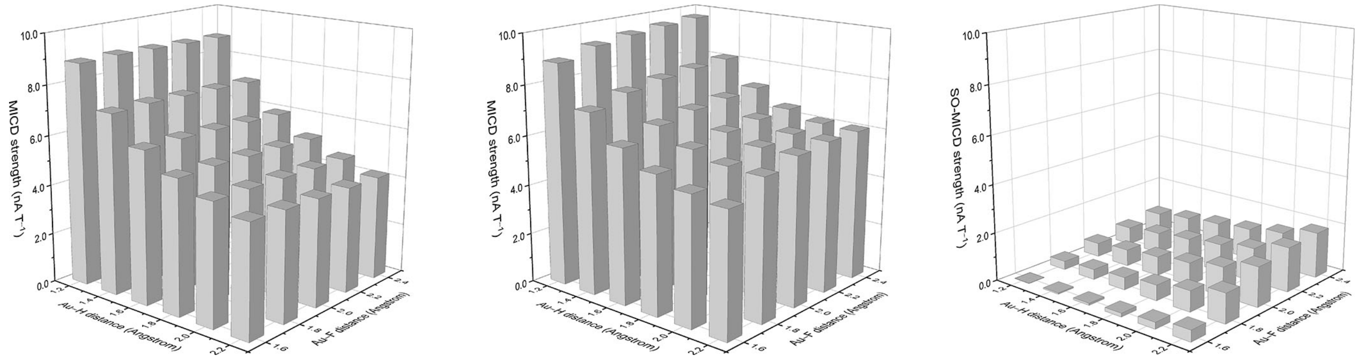

Figure S17: The net MICD strength calculated at the SR-4c-DKS (left) and SOR-4c-DKS (center) levels of theory and the net SO-MICD strength (right), as a function of the Au–H and Au–F distances for molecule **1a**.

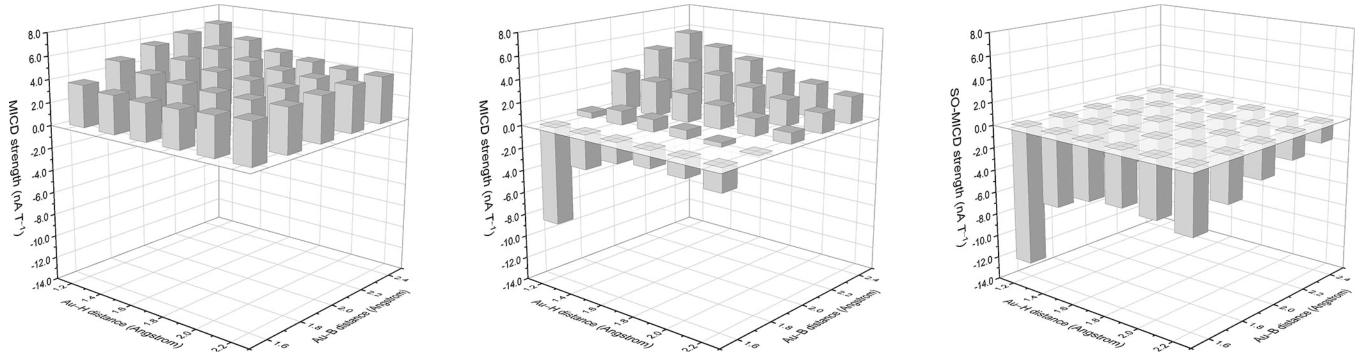

Figure S18: The net MICD strength calculated at the SR-4c-DKS (left) and SOR-4c-DKS (center) levels of theory and the net SO-MICD strength (right), as a function of the Au–H and Au–B distances for molecule **1g**.

Table S30: The energy (in Hartree) and the atomic orbital composition (in %) of the non-relativistic molecular orbitals involved in the leading contributions to $\sigma^{\mathrm{FC/SO}}$ of molecules AuH, **1a**, **1e**, **1f**, and **1g**

Molecule	Label	E	Au AO			H AO	X AO	
			5d	6s	6p	1s	2/3s	2/3p
AuH	σ_{sp}	-0.06853	1.5	37.2	48.4	12.4	-	-
	σ_{ss}	-0.23569	16.3	46.3	4.3	32.8	-	-
	σ_{sd}	-0.44056	76.1	1.5	0.0	21.5	-	-
**1a**	σ^*_{ss}	$+0.27225$	0.0	40.4	50.0	6.4	0.0	1.4
	π^*_{pp}	$+0.17760$	0.0	0.0	97.6	0.0	0.0	2.0
	σ_{ss}	-0.03245	21.5	49.7	5.3	21.7	0.0	1.8
	σ_{sp}	-0.10244	8.5	0.0	5.7	20.6	0.0	64.4
	σ_{sd}	-0.23294	56.0	1.6	0.0	15.7	0.0	24.5
**1e**	σ_{ss}	$+0.00754$	21.9	60.1	0.0	9.0	-	-
	σ_{sp}	$+0.00006$	16.3	46.3	4.3	32.8	-	-
	σ_{sd}	-0.16065	66.3	3.4	0.0	14.9	-	-
**1f**	π^*_{pp}	$+0.14569$	0.0	0.0	87.9	0.0	0.0	8.0
	σ_{sp}	-0.05563	0.0	1.6	18.8	37.9	6.0	27.2
	σ_{ss}	-0.06196	17.3	46.5	0.0	8.2	3.3	21.1
	σ_{sd}	-0.23844	68.6	1.4	0.0	15.2	0.0	0.0
**1g**	$\pi^*_{\mathrm{pp_2}}$	$+0.17064$	0.0	0.0	82.1	0.0	0.0	16.7
	$\pi^*_{\mathrm{pp_1}}$	$+0.14945$	0.0	0.0	102.7	0.0	0.0	-4.3
	π^*_{dp}	$+0.07783$	3.7	0.0	22.2	0.0	0.0	73.2
	σ_{sp}	$+0.01464$	1.9	10.9	12.4	15.4	11.7	40.8
	σ_{ss}	-0.00677	15.0	44.9	7.1	32.5	0.0	0.0
	σ_{sd}	-0.16000	63.0	1.1	0.0	13.0	0.0	13.0
